# Recent Developments in Metallic Degradable Micromotors for Biomedical and Environmental Remediation Applications

**DOI:** 10.1007/s40820-023-01259-3

**Published:** 2023-11-30

**Authors:** Sourav Dutta, Seungmin Noh, Roger Sanchis Gual, Xiangzhong Chen, Salvador Pané, Bradley J. Nelson, Hongsoo Choi

**Affiliations:** 1 Department of Robotics and Mechatronics Engineering, Daegu Gyeongbuk Institute of Science and Technology (DGIST), Daegu, 42988 Republic of Korea; 2 DGIST-ETH Microrobotics Research Center, DGIST, Daegu, 42988 Republic of Korea; 3 Multi-Scale Robotics Lab, Institute of Robotics and Intelligent Systems, ETH Zurich, 8092 Zurich, Switzerland; 4 Institute of Optoelectronics, State Key Laboratory of Photovoltaic Science and Technology, Shanghai Frontiers Science Research Base of Intelligent Optoelectronics and Perception, Fudan University, Shanghai, 200433 People’s Republic of China

**Keywords:** Magnesium, Zinc, Iron, Biodegradable microrobot, Biomedical, Environmental

## Abstract

Synthetic micromotor has gained substantial attention in biomedicine and environmental remediation. Metal-based degradable micromotor composed of magnesium (Mg), zinc (Zn), and iron (Fe) have promise due to their nontoxic fuel-free propulsion, favorable biocompatibility, and safe excretion of degradation products Recent advances in degradable metallic micromotor have shown their fast movement in complex biological media, efficient cargo delivery and favorable biocompatibility. A noteworthy number of degradable metal-based micromotors employ bubble propulsion, utilizing water as fuel to generate hydrogen bubbles. This novel feature has projected degradable metallic micromotors for active in vivo drug delivery applications. In addition, understanding the degradation mechanism of these micromotors is also a key parameter for their design and performance. Its propulsion efficiency and life span govern the overall performance of a degradable metallic micromotor. Here we review the design and recent advancements of metallic degradable micromotors. Furthermore, we describe the controlled degradation, efficient in vivo drug delivery, and built-in acid neutralization capabilities of degradable micromotors with versatile biomedical applications. Moreover, we discuss micromotors’ efficacy in detecting and destroying environmental pollutants. Finally, we address the limitations and future research directions of degradable metallic micromotors.

## Highlights

- This review discusses the potential of degradable metallic micromotors for a variety of biomedical and environmental applications.
- The design principles, fabrication techniques and degradation mechanisms of degradable metallic micromotors are reviewed in detail.
- Challenges and future directions for the development of degradable metallic micromotors for real-life applications are presented.

## Graphical abstract

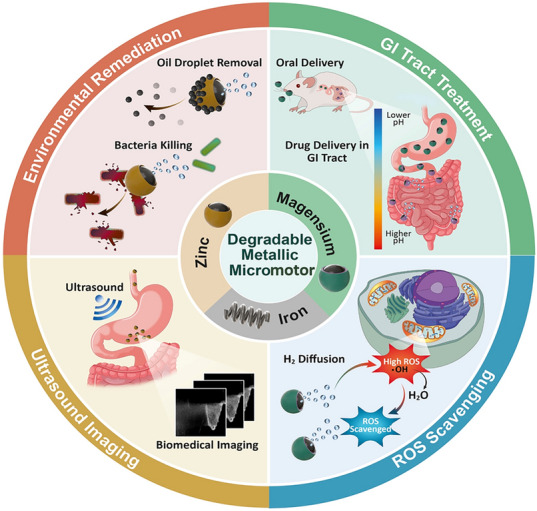

## Introduction

Microorganisms, which are ubiquitous in nature, are equipped with micromachines that enable autonomous propulsion and responses to external stimuli. Inspired by the remarkable properties of these organisms, the concept of microrobots has attracted considerable attention. These microrobots are diminutive synthetic devices that convert external or internal energy sources into motion. Extensive research has been conducted on these devices by multiple research groups in the biomedical and environmental fields due to their many advantages. Microrobots are very promising for biomedicine and can be used for facilitating easy diagnosis, rapid treatment, and speedy recovery from various diseases [1–10]. In addition, microrobots can reduce the invasiveness of treatments and surgeries compared to open surgery, radiation therapy, and manual interventions.

Although microrobots have shown potential for targeted drug delivery, most are non-degradable and raise biocompatibility concerns [11–14]. To address this problem, microrobots may be coated with a layer of gold (Au) or titanium oxide (TiO_2_) to improve their biocompatibility [15–19]. However, these layers are also non-degradable and can lead to chronic inflammatory reactions and accumulation in organs [20, 21]. Therefore, there is a need to develop degradable medical microrobots that can gradually degrade and metabolize a target substance without causing toxicity to surrounding tissues. Researchers have recently focused on developing degradable microrobots using polymers such as poly(lactic glycolic acid) (PLGA) [18, 22], polyethylene glycol (PEG) [23, 24], and gelatin methacryloyl (GELMA) [12]. However, the Food and Drug Administration (FDA) has approved only PLGA for drug delivery applications [25, 26]. In vivo degradation of PLGA involves hydrolysis, which produces glycolic acid and lactic acid as degradation products. This may lead to an acidic environment and an inflammatory response around the target area [27, 28]. Polymeric degradable microrobots are generally incapable of self-propulsion and require non-degradable particles (SPION, Ni etc.) in their structure, powered by an external energy source for targeted delivery [12, 18]. Additionally, real-time observation of biopolymer-based microrobots using X-ray imaging is challenging due to their low density [29, 30]. Metal-based degradable micro-swimmers are a potential alternative due to their excellent biocompatibility, enhanced mechanical properties, and efficient self-propulsion [31, 32]. The movement of these tiny micro-swimmers relies on energy or power sources as the driving force for their locomotion. The specific energy sources (self-propulsion or external sources) depend on the design and materials of the micro-swimmer. In this regard, chemical swimmers are classified as motors because their speed and directionality cannot be controlled. By contrast, swimmers that can be controlled externally are considered robots [33–35]. Thanks to their remarkable locomotion capabilities, micromotors have a wide range of applications, including applications in biomedicine and environmental remediation.

The potential of micromotors to mitigate environmental pollution has attracted a great deal of interest in recent years [36–40]. Traditional remediation methods based on diffusion-based processes require external movement for effective waste treatment [37, 41, 42]. However, self-driving micromotors can efficiently interact with pollutants, thereby overcoming diffusion-limited reactions. These synthetic micro/nanomotors are a promising alternative for environmental remediation. Several research groups have demonstrated the efficacy of micromotors in removing pollutants from the environment using techniques such as the Fenton reaction, activated charcoal absorption, and oxidative detoxification in peroxide solutions [36, 37, 43–49]. However, the need for additional fuels to operate these micromotors can pose environmental and safety risks. To address these concerns, researchers are exploring a new class of microrobots that can harness energy from their surroundings, eliminating the need for additional fuel [31, 50].

Recent reviews on degradable metallic microrobots primarily focus on treating gastrointestinal (GI) tract diseases. However, the fuel-free propulsion and favorable biocompatibility of degradable metallic microrobots have led to multiple biomedical and environmental remediation researches in the past few years. This review provides a comprehensive analysis of recent advances in the development of metallic degradable microrobots for biomedical applications and environmental remediation. The discussion starts by exploring propulsion methods for microrobots, followed by a detailed examination of the characteristics, design principles, fabrication methods and degradation mechanism of degradable metallic microrobots. We also examine the multiple applications of these microrobots in biomedicine and environmental remediation. Finally, we conclude with an overview of the current challenges and future research directions for metallic degradable microrobots.

## Propulsion of Micro/Nanorobots/Motors

First-generation drug carriers relied on passive diffusion and natural body fluid flow for drug delivery [51–53]. The propulsion of micro/nanorobots/motors in a liquid medium is a significant challenge and has been the subject of extensive research. Several active propulsion techniques have been developed that allow micro/nanorobots/motors to outperform the natural flow of body fluids and ultimately improve targeting efficiency in anatomically challenging body regions such as the brain, liver, and eye [5, 12, 16, 54, 55].

The motion of micro/nanoparticles (NPs) is mainly governed by viscous surface forces that dominate their inertial effect, necessitating the use of nonlinear motion mechanisms for successful propulsion at low Reynolds numbers in microscale environments [51–59]. In addition, their small size limits the power supply, making the drive even more demanding. Power for the microrobot can be classified as local (*e*.*g*., chemical and biohybrid designs), external (including magnetic, acoustic, light and electrical sources), or a combination of both.

### Magnetic Propulsion

**Fig. 1:**
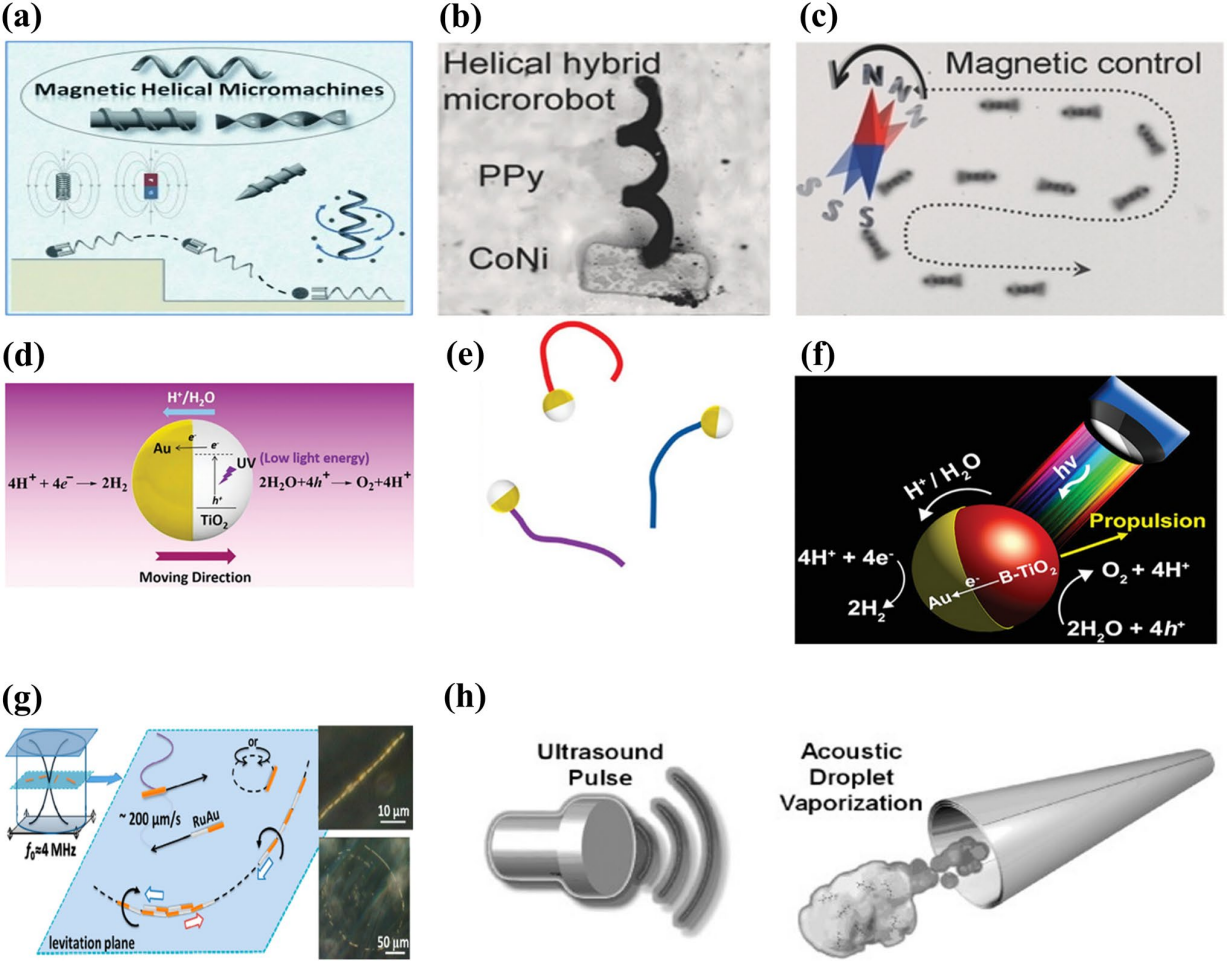
Schematic representation of (**a**) magnetic actuation. Reproduced with permission [70]. Copyright 2013, Wiley Publication. (**b**) Helical hybrid magnetic microrobot. Reproduced with permission [67]. Copyright 2014, Wiley Publication. (**c**) Actuation of a helical hybrid microrobot under magnetic field. Reproduced with permission [67]. Copyright 2014, Wiley Publication. (**d**) Catalytic TiO_2_–Au Janus micromotor powered by UV light. Reproduced with permission [77]. Copyright 2016, American Chemical Society. (**e**) Trajectory of light-powered micromotors. Reproduced with permission [77]. Copyright 2016, American Chemical Society. (**f**) Light-responsive Janus B-TiO_2_/Au micromotor. Reproduced with permission [80]. Copyright 2017, American Chemical Society. (**g**) Trajectory of an asymmetric metallic microrod by self-acoustophoresis. Reproduced with permission [86]. Copyright 2012, American Chemical Society. (**h**) Bubble recoil propulsion of a metallic microtube under ultrasound. Reproduced with permission [87]. Copyright 2012, Wiley Publication

The magnetic propulsion of micro/nanorobots is promising due to its small-scale remote-control capability. Magnetically controlled micro/nanorobots can experience rotating magnetic torques or pulling magnetic forces using a permanent magnet or an electromagnetic coil. Typically, magnetic materials such as superparamagnetic iron oxide nanoparticles (SPIONs), nickel, iron [Fe], or cobalt are incorporated into the micro/nanorobot structure along with optimized magnetic fields to achieve the desired locomotion [60–65]. Various spiral magnetic microrobots inspired by the movement of *Escherichia coli* bacteria have been developed for drug delivery applications [23, 64, 66–70]. These microrobots spin around their main axis under a rotating magnetic field, generating a corkscrew motion that converts magnetic force into mechanical actuation of the microrobot (Fig. 1a–c) [17, 66, 70]. Consequently, helical microrobots containing magnetic particles can propel themselves by rotation or oscillation instead of being pulled by an external magnetic field. By contrast, magnetic walkers or drum-based micro-robots roll on a surface during propulsion and maintain constant interaction with the surface [71, 72]. However, successful clinical implementation of magnetically actuated microrobots is constrained by the need for specialized equipment (stereotaxis) and a limited workspace.

### Light-Driven Propulsion

The use of light as a physical stimulus to facilitate the movement of micro/nanomotors is an established concept. Various materials have been developed that can convert energy from different regions of the electromagnetic spectrum into mechanical work. The response of a light-powered micro/nanomotor to light irradiation depends on its photoactivity. Such motors can be classified as photocatalytic, photothermal, photochromic, or photosensitive.

Photocatalytic materials have been studied for their advantages, such as efficient propulsion, fast response to light stimuli, and activation by a wide range of wavelengths [73–76]. Examples of photocatalytic materials are metal oxide semiconductors such as titania, silica, bismuth oxide, and oxometallates [77–81]. When these materials are activated by ultraviolet (UV), visible, or infrared radiation, the energy provided promotes an electron from the valence band to the conduction band. The result is an electro-photochemical reaction based on the wavelength of the incident light and the micro-/nanomotor material (Fig. 1d–f).

Photothermal materials such as carbon, silica, and polymers containing gold nanoparticles (AuNPs) are also light-powered micro/nanomotors that convert light energy into thermal energy [82–85]. By inducing asymmetric preferential absorption of near-infrared (NIR) light, photothermal micro/nanomotors create a thermal gradient across the surface of the particle and the surrounding solution. These motors offer a high level of tunability and can operate over a wide range of the electromagnetic spectrum. However, the performance of current-generation micro/nanomotors is limited by the penetration of light in biological samples.

### Acoustic Propulsion

Acoustic propulsion is a promising approach for externally powering micro/nanomotors due to its high tissue penetration, tunability, and biocompatibility. As sound waves propagate through a liquid medium, they generate a forward pressure that propels micro/nanomotors. Piezoelectric transducers are typically used to generate surface acoustic and ultrasonic propulsion sound waves. Ultrasonic propulsion has attracted much attention in micro/nanorobotics. The waves generated can be classified as standing, travelling, or focused ultrasound waves.

First-generation ultrasonic micro/nanomotors were powered using a self-acoustophoresis mechanism due to the shape asymmetry of the metallic nanowire (Fig. 1g) [86]. However, their movement was limited to the small area around the acoustic nodes. Micro/nanomotors also use acoustic travelling waves for actuation, where the travelling wave-induced vibration of the bubble enclosed in the micro/nanomotor structure enables its movement in the liquid medium. For example, Kagan et al. reported evaporative propulsion of tubular microrobots by high-intensity focused ultrasound [87] (Fig. 1h). The tubular micromotors receive a sudden impulse and reach a speed of 6.3 m s^−1^ due to rapid evaporation of the chemical fuel-perfluorocarbon emulsion.

Focused ultrasound is a safe, inexpensive, and effective method for delivering drug molecules to various tissues [88, 89]. However, successful development of ultrasonically controlled micro/nanomotors depends on the choice of materials. A high-density contrast between the constituent material of micro/nanomotors and surrounding fluid is necessary for strong propulsion [90]. For instance, micro/nanostructures in which gas microbubbles are embedded exhibit a high-density contrast with their surrounding medium, enabling strong propulsion upon application of an acoustic field [91, 92].

### Chemical Propulsion

Chemical propulsion is a promising approach for localized actuation of micromotors. Micromotors can harness chemical energy from their environment via specific chemical reactions. Chemically active materials, such as noble metals (*e.g*., platinum [Pt], Au, and silver [Ag]), transient metals (*e.g*., Fe, zinc [Zn], and magnesium [Mg]), metal alloys, reactive materials (*e.g*., calcium carbonate), and bio-catalytic enzymes (*e.g*., urease, catalase, and glucose oxidase), have been used for this purpose [46, 56, 93–96]. By incorporating these materials into micro/nanostructures in combination with a green fuel, propulsion can be achieved.

**Fig. 2:**
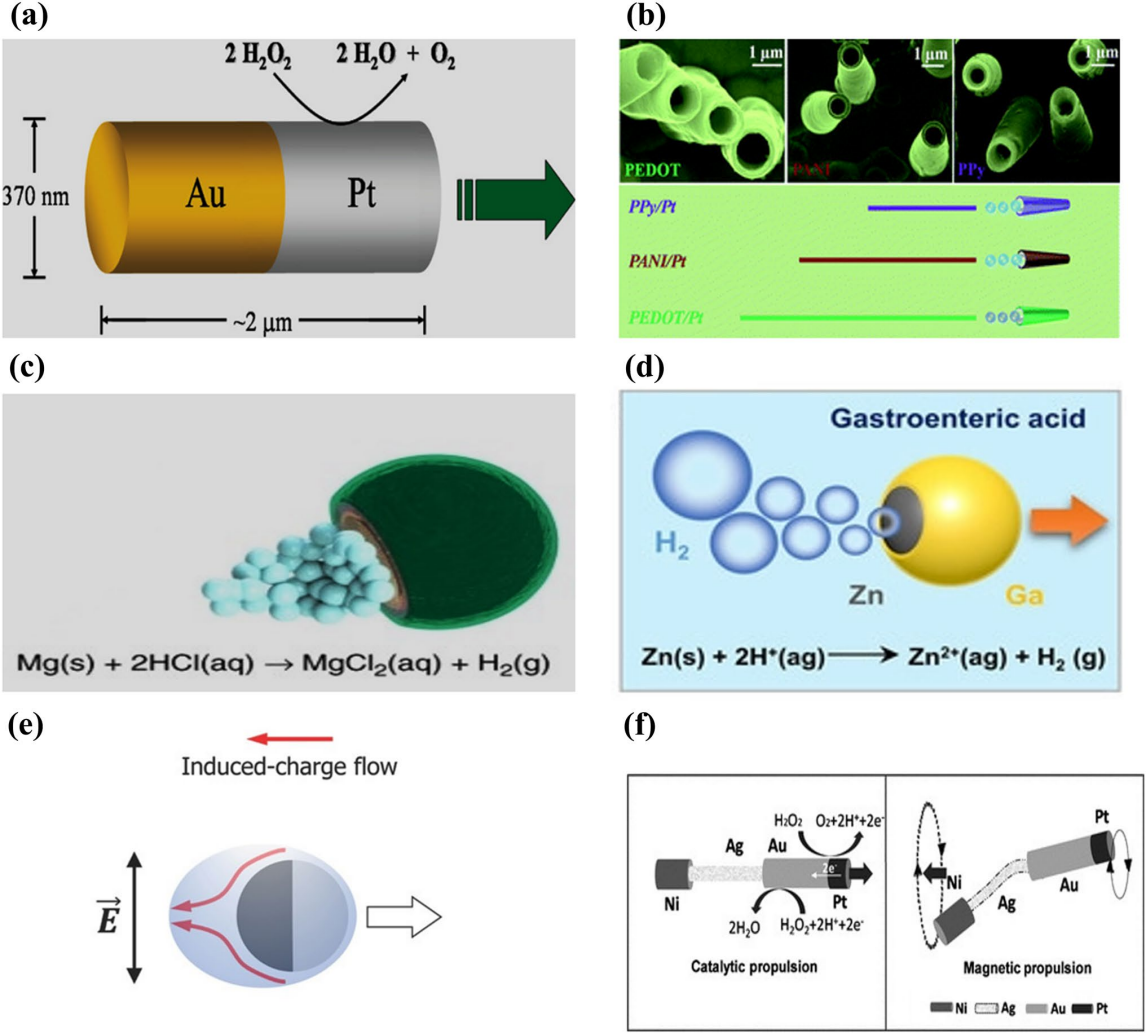
Schematic representation of (**a**) asymmetric catalytic propulsion of an Au-Pt micromotor. Reproduced with permission [97]. Copyright 2004, American Chemical Society. (**b**) Directional motion of a polymer-based bilayer microtube in 5% H_2_O_2_ solution. Reproduced with permission [102]. Copyright 2012, Royal Society of Chemistry. (**c**) Chemical propulsion of an Mg-based micromotor in an acidic environment. Reproduced with permission [144]. Copyright 2017, Springer Nature. (**d**) Chemical propulsion of a Zn-based micromotor in gastric acid. Reproduced with permission [223]. Copyright 2021, Wiley Publication. (**e**) Electric field induced propulsion of Janus particle under AC field. Reproduced with permission [110]. Copyright 2017, Wiley Publication. (**f**) Hybrid microrobot propelled by catalytic and magnetic propulsion. Reproduced with permission [113]. Copyright 2011, Wiley Publication

Bimetallic nanowires of Au and Pt have been developed as early-stage catalytic engines and immersed in hydrogen peroxide fuel [97]. The electrocatalytic decomposition of the hydrogen peroxide fuel results in an asymmetric ion distribution around the bimetallic nanowire, generating an electrokinetic flow in the vicinity of the nanowire and leading to its movement (Fig. 2a). However, these catalytic micromotors require a hydrogen peroxide environment, which might limit their real-life applications. In addition, the thickness of the electrical double layer around the motor governs its velocity and is adversely affected at high salt concentrations [56, 98]. Chemically powered catalytic micro/nanomotors also have extremely low energy efficiencies in the order of 10^−8^–10^−9^.

Another alternative approach to locally powered chemical propulsion is continuous bubble emission from micro/nanomotors. This mechanism generates greater velocity and power compared to self-electrophoresis and self-diffusiophoresis [99–101]. The catalytic surface of bubble-powered micro/nanomotors is encapsulated by inert materials such as silicon, perylene, polypyrrole, and graphene, leaving a small opening for the fuel to react with the motors [102–104]. It helps to achieve directional motion for the catalytic micro/nanomotors (Fig. 2b). Furthermore, micro/nanomotors based on Janus microspheres with a diverse range of openings, including half coating or nearly complete coating with a small hole, are also used for bubble propulsion (Fig. 2c–d). For instance, Mg was coated with an Au layer and a small opening was left in the microrobot [105]. When immersed in a physiological solution, hydrogen emission triggered propulsion due to macro-galvanic corrosion. The efficiency of micro/nanomotor propulsion depends on the frequency of bubble production, which is governed by the material, the surface roughness, and the geometric design of the micro/nanomotor. However, the use of toxic chemicals such as hydrogen peroxide, hydrazine, and sodium borohydride, as fuels for chemical propulsion hinders their biomedical application [106–109].

### Electric Propulsion

An external electric field is also widely used for microrobot propulsion. An external electric field polarizes particles, which accumulate countercharges in the electric double layer (EDL) surrounding the particle [56, 110]. Subsequently, the applied field influences the induced charges to initiate electroosmotic flow. The more and less polarizable halves of Janus-type particles result in active propulsion under an alternating current (AC) field. Successful electric propulsion requires a combination of materials with distinct electric susceptibility. For example, metallic materials are considerably more strongly polarized than dielectric materials. When an electric field is applied, the metallic side experiences more polarization than the dielectric side [110]. Consequently, the microrobot propels itself with the dielectric side oriented forward due to the strong induced charge electro-osmosis (ICEO) around its metallic region [111]. The overall mechanism relies on generating asymmetric flow through the induced charges around the microrobot, as shown in Fig. 2e. When these microrobots are subjected to a uniform direct current (DC) field, they exhibit dipole behavior [112]. As a result, the charged end moves toward the opposite electrode through the contact charge electrophoresis (CCEP). In general, an AC electric field is applied to control the microrobot motion, as opposed to utilizing DC fields, thereby eliminating the electrophoresis effect.

### Hybrid Propulsion

Local power-based microrobots are beneficial for carrying cargo and enhanced mixing. The direction of the microrobot is determined by itself and its environment. By contrast, externally powered microrobots offer tunable on-demand propulsion. To harness the advantages of both these techniques, the concept of a microrobot equipped with multiple engines is introduced. For example, a microrobot utilizing chemical and magnetic propulsion techniques has a catalytic head and flexible magnetic tail [113] (Fig. 2f). Initially, the microrobot moves solely via a chemical reaction. When the fuel is exhausted, an external magnetic field is applied to control its actuation, which is achieved by oscillating the flexible tail. A similar design was used to develop chemical/light and magneto-acoustic microrobots [114, 115]. For example, a magneto-acoustic microrobot had a helical structure coupled with a concave gold segment [115]. A microrobot with this type of twin power source has finely tunable propulsion. Furthermore, the hybrid propulsion technique may open-up new avenues of motion control with different modes (accelerating or braking) that a single engine could not achieve.

Biohybrid microrobots have recently been considered promising candidates for biomedical applications. These microrobots contain both biological and non-biological materials [116, 117]. For instance, magnetic particles have been embedded in microalgae (e.g., Spirulina, Chlorella, and diatoms) and some bacteria (e.g., E. coli and MTB) to develop such microrobots [118–122]. Biohybrid microrobots provide high flexibility and excellent driving force due to the presence of living tissues. The helical structures of magnetic microrobots enable efficient locomotion using cork-screw propulsion. Using Spirulina as a substrate, the bio-templated fabrication technique yields these helical structures, establishing it as the favored optimal approach [119]. Furthermore, different bodily cells (stem cells, red blood cells (RBC), and macrophages) have also been used to develop biohybrid magnetic microrobots due to their ability to transport therapeutic payloads and respond to biochemical signals [123]. Incorporating magnetic nanoparticles onto such biohybrid microrobots enables them to respond under an external magnetic field, facilitating precise navigation to hard-to-reach regions in the human body.

## Metallic Biodegradable Micromotor

Early micro/nanorobots predominantly relied on non-degradable materials. After a treatment, these micro/nanorobots should be excreted from the body within a specific time-period. However, the natural excretion of microrobots depends on various parameters such as design, size, concentration of dosage, etc. For example, magnetic microrobots are widely used for targeted drug delivery purposes. Recent studies indicate that magnetic nanoparticles (MNPs) larger than 100 nm can be excreted through the renal pathway within a limited time [124, 125]. If these particles are not completely excreted from the body, they may accumulate in the vital organs such as the liver, kidney or heart, and lead to chronic inflammatory responses. For instance, 55% of the intravenously injected oleic acid/pluronic-coated MNPs accumulated in the rat liver [126]. The accumulation of MNPs can potentially lead to oxidative stress, DNA damage, epigenetic events, and inflammatory processes [127–129]. Therefore, non-degradable microrobots may result in acute or chronic toxicity and require surgical intervention for removal [18, 130, 131]. In addition, retrieving these microrobots once their task is complete is challenging. Such non-degradable robots are typically rigid and hard, potentially leading to tissue injury and blood vessel rupture [132]. These challenges of non-degradable microrobots may significantly reduce their effectiveness.

Biodegradation is a time-dependent decomposition process facilitated by biological activities. It is a critical design parameter for microrobots, as these devices are expected to degrade into nontoxic metabolites after they have served their intended function. While synthetic polymers such as PEG, PLGA, and GELMA have been investigated extensively as degradable microrobots, their acidic degradation products have impeded their practical application [133, 134]. By contrast, the degradation products of natural polymers do not elicit a toxic response [135, 136], but their inferior processability typically allows the creation of large scaffolds by the mold-casting method [136, 137]. Therefore, a degradable microrobot needs a balance between degradability and mechanical strength. However, the majority of the materials used for microrobots display noncomplementary mechanical strength and degradation behaviors.

Trace elements such as Mg, Zn, and Fe, and their alloys, can be degraded in vivo via chemical reactions in physiological environments. The degradation products of these materials can easily be excreted without causing a toxic response [138, 139]. The three different metallic degradable micromotors are compared in Table 1. Moreover, these materials have enhanced mechanical properties and are degraded more rapidly than synthetic polymers. By contrast, catalytic non-degradable micromotors are typically employed for environmental remediation. However, these robots rely on toxic external fuel and decontamination reagents, leading to incomplete remediation and impeding their practical application. Therefore, self-propelled Janus-type noncatalytic metallic degradable micromotors may be an alternative approach to facilitate environmental remediation.

**Table 1:** Comparison of different parameters, advantages and disadvantages of Mg/Zn/Fe micromotors

	Mg based	Zn based	Fe based
Daily intake (mg/day)	400	4–14	18
Degradation time	Faster	Moderate	Low
Actuation technique	Chemical	Chemical	Magnetic
Velocity	Uncontrollable	Uncontrollable	Controllable
Biocompatibility	Excellent	Moderate	Low
Advantages	(i) High daily intake	(i) Degradation rate is lower than Mg	(i) Slow degradation
	(ii) Excellent biocompatibility	(ii) Moderate biocompatibility	(ii) Velocity can be controlled
	(iii) No external power source is required for propulsion	(iii) No external power source is required for propulsion	
Disadvantages	(i) Faster degradation rate	(i) Lower daily intake	(i) Low daily intake
	(ii) Uncontrollable motion	(ii) Uncontrollable motion	(ii) Low biocompatibility
			(iii) External source is required for motion control
Potential applications	Biomedical, environmental	Biomedical	Biomedical
Refs.	[31, 144, 171]	[140, 223, 230]	[141, 231]

## Design Philosophy of Degradable Metallic Micromotors

**Fig. 3:**
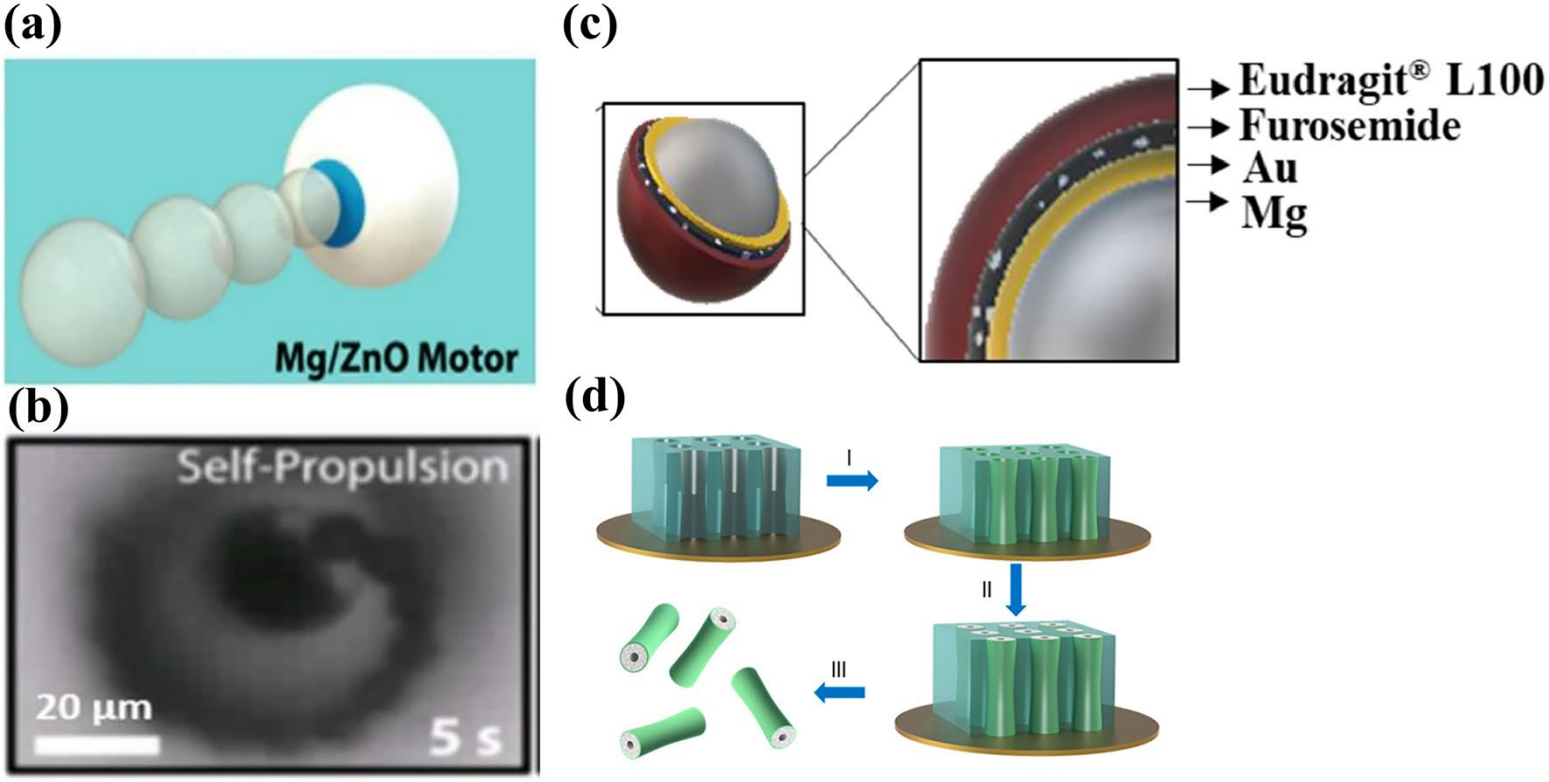
Schematic representation of (**a**) self-propelled Mg-ZnO micromotor. Reproduced with permission [140]. Copyright 2016, American Chemical Society. (**b**) Self-propulsion of Mg-ZnO micromotor under optical microscope. Reproduced with permission. [140]. Copyright 2016, American Chemical Society. (**c**) Schematic of an Mg/Au/Furosemide/polymer-coated micromotor. Reproduced with permission [146]. Copyright 2022, Elsevier. (**d**) Representative image of template-assisted fabrication of a Zn micromotor. Reproduced with permission [225]. Copyright 2015, American Chemical Society

The design of degradable metallic micromotors for biomedical applications requires careful consideration of material selection and fabrication techniques. In particular, the self-propelled devices have to perform their intended task, such as drug delivery or environmental remediation, and subsequently degrade naturally without causing any harm to the surrounding tissue or environment. Two categories of these micromotors/robots are spherical Janus particles and three-dimensional structures, such as helix [140, 141]. In particular, self-propelled Janus particles have demonstrated promising results as the most degradable metallic micromotors (Fig. 3a, b).

To fabricate Janus particles, noble metal (Au/Pt/Ag) and drug layers are deposited onto spherical Mg particles. The formation of a passive Mg hydroxide layer upon exposure to water at ambient temperature prevents Janus-type Mg micromotors from using hydrogen bubble propulsion in water. However, these micromotors work efficiently in chloride-rich environments, such as body fluids or seawater, via macro-galvanic corrosion and chloride-pitting corrosion [31]. In such environments, Mg continuously reduces water, creating hydrogen bubbles for propulsion. The dissolution rate of Mg in chlorine-rich water depends on the electrochemical potential difference between Mg and the deposited metal. For instance, the standard electrode potentials of Ag and Au are + 0.80 and + 1.50 V, respectively, whereas Mg has a very low standard electrode potential of − 2.37 V. As a result, the difference in standard electrode potential for Au-Mg systems is higher, leading to considerably faster degradation of Mg compared to Ag-Mg systems. Therefore, the choice of deposited layer material can markedly affect the lifespan of Janus-type Mg-based micromotors.

One of the significant challenges in designing degradable metallic micromotors is their short lifespan, which may limit their ability to complete their task. Their degradation rate highly influences the performance of these micromotors. A faster degradation rate always hinders them from completing their assigned task. Upon exposure to the body fluid, these micromotors immediately react because of their high reactivity. As a result, early exposure to body fluid can compromise their performance. To address this limitation, micromotors may be encapsulated in a carrier or microcapsule that unloads them upon reaching the target area. For example, enteric gelatin capsules, lactose and maltose, PLGA etc. could be used as carrier or microcapsule [142, 143]. This strategy can prolong the lifespan of degradable metallic micromotors and increase their functional efficiency.

## Fabrication of Metallic Degradable Micromotors

Fabricating metallic degradable micromotors is challenging due to their high reactivity in the atmosphere. Metallic degradable micromotors can be fabricated using two distinct methods: onion-like layer-by-layer deposition on a glass slide and electrochemical processes [140, 142]. In the following section, several fabrication techniques for developing degradable metallic micromotors are discussed.

### Onion-Like Layer-by-Layer Deposition for Metallic Degradable Micromotors

Most of the literature on the fabrication of metal-based degradable micromotors has focused on onion-like layer-by-layer deposition on a glass slide because of its simplicity and low-cost involvement. The fabrication technique described in this section involves layer-by-layer deposition using powder particles and sputtering of noble metals (Au/Ag/Pt) or TiO_2_ onto their surface on a glass slide [144–146]. This fabrication technique generates a tiny opening in the particle-glass interface that enables the micromotor core to interact with the surrounding medium and achieve bubble propulsion [147]. After the deposition of the metal/ceramic oxide layer, a separate layer containing a drug molecule is deposited. Figure 3c shows drug-loaded Mg-based micromotors. Despite the successful development of degradable metallic micromotors using this fabrication technique, rapid degradation is a significant concern. Therefore, a degradable metallic micromotor carrier is proposed [142, 143]. It has an enteric coating that degrades upon reaching the target site, releasing the degradable metallic micromotors to perform their designated functions [143].

### Electrochemical Processes

Electrochemical deposition is a versatile and cost-effective method for fabricating micro/nanostructures with controlled shapes [148]. This method is based on the reduction of metal ions from aqueous, organic, or ionic liquid electrolytes by an external electrical field, causing them to deposit onto conductive substrates. The kinetics and mechanisms of the electrochemical reaction at the metal-solution interface, as well as the nucleation and growth processes of the metal lattice, determine the size of the resulting micro/nanostructures [149].

$${\text{M}}^{{\text{n + }}} + {\text{ne}}^{ - } \to {\text{M}}_{{\text{(s)}}}$$

A typical three-electrode deposition system consists of an electrochemical bath, two electrodes (working and counter electrodes), and one reference electrode. The electrochemical bath contains a solution of metallic ions to be deposited on the working electrode, where reduction mainly occurs, while oxidation typically takes place on the counter or reference electrode. Cathodic deposition of metal ions is preferred due to their positive charge, whereas anodic deposition is rarely employed due to its poor adhesion and stoichiometric composition.

Template-based fabrication is used for the electrodeposition of micromotors, where a negative replica of a rigid template is developed to produce exact shapes. This technique offers an easy way to fabricate complex micro/nanostructures such as nanowires, nanotubes, micro-helices, and porous nanocones (Fig. 3d). The electrodeposition method is simple, scalable, and versatile, making it a promising approach for various applications in nanotechnology and microengineering.

## Degradation Mechanism of Metallic Degradable Micromotor

Degradable metallic micromotors such as Mg, Zn, and Fe are made from reactive metals. These materials react easily with water or acid, resulting in hydrogen bubble formation. In general, Janus-type degradable metallic micromotors are propelled by a chemical reaction that eventually leads to their degradation [31, 144]. The degradation of these micromotors is governed by macro-galvanic corrosion and the presence of anions in the medium [50, 146, 150].

**Fig. 4:**
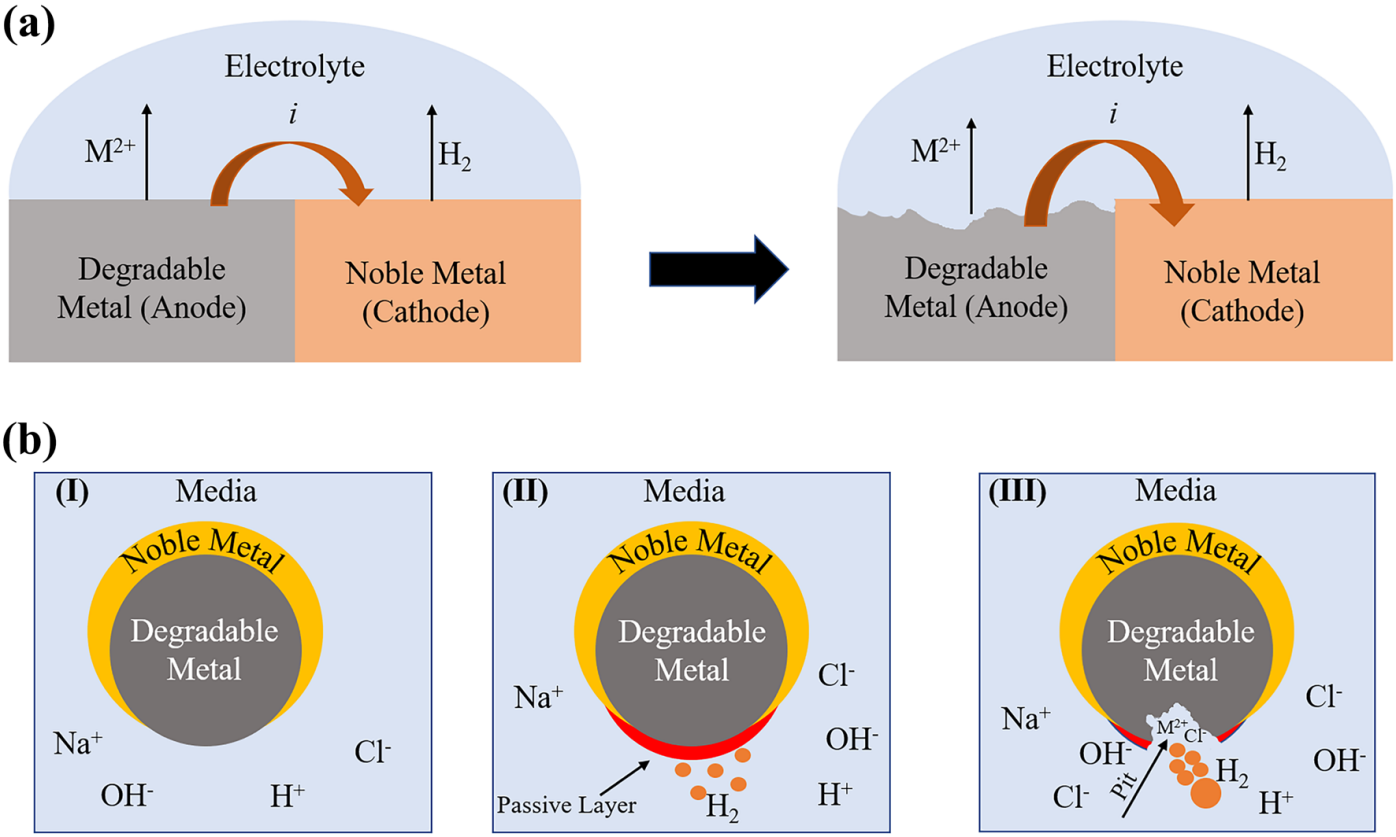
(**a**) Schematic of macro-galvanic corrosion. (**b**) Degradation mechanism of a degradable metallic microrobot in chloride ion-rich medium. (I) Microrobot just after exposure to medium, (II) passive layer formation on the surface, (III) aggressive anion (chloride)-induced pitting corrosion

Macro-galvanic corrosion is an electrochemical reaction between two dissimilar metals that are in electrical contact [151]. One of them acts as a cathode, and the other one acts as an anode due to the difference in standard electrode potential. A schematic of macro-galvanic corrosion is shown in Fig. 4a. Janus-type degradable metallic micromotors are designed to make a macro-galvanic electrochemical cell. For example, noble metals (Au/Pt) are deposited on the surface of degradable metals [50, 143, 152]. As a result, it forms a macro-galvanic cell in the presence of surrounding media. When dispersed in chloride ion rich water, these particles react with the medium to form a metal hydroxide on the surface. For example, a magnesium hydroxide layer rapidly forms on the Mg surface of Mg/Pt Janus particles exposed to blood plasma or human serum according to the following reaction [153]:

1 $${\text{Mg}} \to {\text{Mg}}^{{2 + }} + 2{\text{e}}^{ - }$$

2 $${\text{2H}}_{{2}} {\text{O}} + 2{\text{e}}^{ - } \to 2{\text{OH}}^{ - } + {\text{H}}_{2} \left( {\text{g}} \right)$$

3 $${\text{Mg}}\,\left( {\text{s}} \right) + {\text{2H}}_{{2}} {\text{O}} \to {\text{Mg}}\left( {{\text{OH}}} \right)_{{2}} \left( {\text{s}} \right) + {\text{H}}_{{2}} \left( {\text{g}} \right)$$

Body fluid is also rich in chloride ions [154, 155]. The degradation mechanism of degradable metallic micromotors is significantly influenced by this ion. Although the hydroxide layer is stable in water, chloride ions in the solution significantly accelerate macro-galvanic corrosion. For example, upon contact with blood plasma or human serum, the passive hydroxide layer reacts with chloride ions to produce highly soluble magnesium chloride (MgCl_2_) [50]:

4 $${\text{Mg}} + 2{\text{Cl}}^{ - } \to {\text{MgCl}}_{{2}}$$

5 $${\text{Mg}}\left( {{\text{OH}}} \right)_{{2}} \,\left( {\text{s}} \right) + 2{\text{Cl}}^{ - } \,\left( {{\text{aq}}} \right) \to {\text{MgCl}}_{2} + {\text{H}}_{{2}} \left( {\text{g}} \right)$$

In general, pitting corrosion happens in metals with a protective passive oxide layer [50, 156, 157]. Aggressive anions like chloride can easily penetrate the passive layer and enable their electrostatic transport within the pit. Furthermore, the charge balance in the corrosion pits is maintained by an increase in the Mg^2+^ ion concentration. As corrosion progresses, the pit surface experiences OH depletion, which hinders its passivation. The pit environment becomes mildly acidic because of the accumulation of Mg^2+^ and Cl^−^ ions, further promoting the degradation of Mg. Therefore, the synergistic effect of macro-galvanic and pitting corrosion enhances the dissolution of degradable micromotors and propulsion. The degradation mechanism is shown schematically in Fig. 4b.

The corrosion of pure Zn progresses through the anodic dissolution of Zn, while oxygen reduction occurs as the dominant cathodic reduction during the initial immersion period according to Eqs. (6) and (7). As the reaction proceeds with time, increases in the Zn^2+^ and OH^−^ concentrations lead to the precipitation of zinc hydroxide (Zn(OH)_2_). Zn(OH)_2_ changes into more thermodynamically stable zinc oxide (ZnO), forming a protective layer on the metal surface to prevent further corrosion. Aggressive anions like Cl^−^ can react with these corrosion products and convert into soluble zinc chloride (ZnCl_2_) [158, 159]. Consequently, the protective layer becomes damaged, leading to rapid degradation.

6 $${\text{Zn}} \to {\text{Zn}}^{2 + } + 2{\text{e}}^{ - }$$

7 $${\text{O}}_{{2}} + {\text{H}}_{{2}} {\text{O}} + 4{\text{e}}^{ - } \to 4{\text{OH}}^{ - }$$

8 $${\text{Zn}}^{{2 + }} + 2{\text{OH}}^{ - } \to {\text{Zn}}\left( {{\text{OH}}} \right)_{2}$$

9 $${\text{Zn}}\left( {{\text{OH}}} \right)_{{2}} \to {\text{ZnO}} + {\text{H}}_{{2}} {\text{O}}$$

10 $${\text{Zn}}^{{2 + }} + {\text{2Cl}}^{ - } \to {\text{ZnCl}}_{2}$$

Zn-based micromotors are also designed as Janus-type particles. Therefore, their degradation is also governed by macro-galvanic corrosion. Most of the Zn-based micromotors were developed for gastrointestinal (GI) tract treatments. For example, the Zn/Fe Janus micromotor was exposed to simulated gastric acid (pH 1.1). The respective anodic indices of Fe and Zn are − 0.75 and − 1.25 V [140]. As a result, it forms a short-circuited galvanic cell when in contact with gastric acid. The Zn core reacts with an acidic solution and releases H_2_ bubble to initiate micromotor propulsion according to the following equation [140]:

11 $${\text{Zn}} + {\text{2HCl}} \to {\text{ZnCl}}_{2} + {\text{H}}_{2}$$

Pure Fe is the most noble of the three biodegradable metals, and its standard electrode potential is − 0.44 V (vs. SHE) [160]. Therefore, it has a slow degradation rate compared to Mg and Zn. The degradation mechanism consists of the anodic dissolution of Fe and cathodic reduction of dissolved oxygen, as shown in Eqs. 12 and 13 [160]. The reaction between the released metal ions and hydroxyl ions leads to the formation of ferrous hydroxide (Fe(OH)_2_) or hydrous ferrous oxide (FeO·nH_2_O). At the outer surface of the hydroxide layer, ferrous (Fe^2+^) oxides are converted into ferric oxide (Fe^3+^) or ferric hydroxide in the presence of dissolved oxygen [161]. Hydrous ferric oxide is brown and the most visible corrosion product. The corrosion products are generally porous and do not cover metal surfaces homogeneously. Hence, Cl^−^ ions diffuse toward the metal surface, lowering the (Fe^2+^) ion concentration and forming ferrous chloride (FeCl_2_). FeCl_2_ is further hydrolyzed by water, generating acid according to Eq. 16 and resulting in localized corrosion [162].

12 $${\text{Fe}} \to {\text{Fe}}^{2 + } + 2{\text{e}}^{ - }$$

13 $${\text{O}}_{{2}} + {\text{H}}_{{2}} {\text{O}} + 4{\text{e}} \to 4{\text{OH}}^{ - }$$

14 $${\text{Fe}}^{{2 + }} + 2{\text{OH}}^{ - } \to {\text{Fe}}\left( {{\text{OH}}} \right)_{{2}}$$

15 $${\text{Fe}}^{{2 + }} + 2{\text{Cl}}^{ - } \to {\text{FeCl}}_{2}$$

16 $${\text{FeCl}}_{{2}} + {\text{H}}_{{2}} {\text{O}} \to {\text{Fe}}\left( {{\text{OH}}} \right)_{{2}} + {\text{HCl}}$$

The degradation of Janus-type Mg and Zn-based micromotors is governed by macro-galvanic corrosion. This phenomenon strongly depends on the difference between the standard electrode potential (E^0^) of the core and that of the deposited noble metal layer. The standard electrode potentials of Mg and Zn are − 2.37 and − 0.76 V, respectively. If a noble metal (Ag, E^0^ =  + 0.8 V) is deposited on both Mg and Zn particles, the degradation rate of the Mg-based micromotor would be higher than that of the Zn-based micromotor due to the large difference in standard electrode potential. Therefore, Zn-based Janus-type micromotors have a longer lifespan than Mg-based micromotors for the same noble metal deposition. In comparison, Fe-based microrobots were fabricated with a 3D helical/roller structure. These degraded slowly in gastric acid fluid, compared to Mg and Zn-based micromotors, because of their higher standard electrode potential.

## Magnesium-Based Micromotors

Mg is an abundant element, ranking eighth in the Earth's crust [163]. As a group II element, it is highly reactive and quickly reacts with water at ambient temperature. In its pure form, Mg does not exhibit desirable mechanical properties compared to other engineering metals, limiting its structural applications. However, Mg shows favorable biocompatibility and degradation in physiological solutions. For instance, Mg alloy-based cardiovascular stents are more advantageous than those of Ti alloy or stainless steel due to their low thrombogenicity, biocompatibility, and degradability [164–166]. Additionally, Mg alloy-based implants, such as bone plates and screws, promote the formation of new bone growth by stimulating osteoblast activity [167–169].

In the body, Mg is the fourth most abundant cation [170–172] with a daily requirement of 400 mg/day [173, 174]. It acts as a cofactor for around 300 enzymes and participates in several metabolic processes [175, 176]. Mg-rich water may reduce the incidence of cardiovascular diseases [177]. The Mg concentration in extracellular fluid ranges from 0.7 to 1.05 mmol L^−1^, and its homeostasis is maintained by the kidneys and intestine [178]. Hyper-magnesium, which can cause muscular paralysis, respiratory distress, and cardiac arrest, occurs when the Mg concentration exceeds 1.05 mmol L^−1^. However, hyper-magnesium is rare due to the efficient excretion of Mg in urine. Conversely, Mg deficiency leads to congestive heart failure, osteoporosis, fibromyalgia, and other diseases [179, 180]. Thus, Mg is suitable for micromotors because of its biochemical and biological advantages.

### Biomedical Applications

#### Gastrointestinal Tract

Oral drug delivery is a convenient method of drug administration due to its non-invasiveness, cost-effectiveness, and high patient compliance. However, it has limitations, such as enzymatic degradation causing the first-pass effect, poor permeability, wide pH variations in the GI tract, and continuous mucus secretion [181]. The GI tract shows marked pH variation, with gastric acid being highly acidic (pH 1–3) and the intestine mildly acidic (pH 6–7). Conventional micro/nanoparticle-based drug delivery systems do not function efficiently in this acidic environment due to a short intestinal retention time, leading to inadequate drug absorption [182, 183]. Therefore, researchers have aimed to develop enzyme-assisted and diffusion-based drug delivery systems [184, 185]. However, these systems have low precision, size restrictions, and specific surface chemistries.

In contrast, in an acidic environment, Janus-type metallic micromotors such as Mg are propelled by the bubble recoil mechanism, regardless of pH [186]. *Helicobacter pylori (H-pylori)* causes stomach infections and other gastric diseases [187, 188]. Antibiotics combined with proton pump inhibitors (PPIs) are the conventional treatment for *H-pylori* infections [189, 190]. However, long-term use of PPIs can lead to various side effects such as headaches, anxiety, depression, and diarrhea [191–194].

**Fig. 5:**
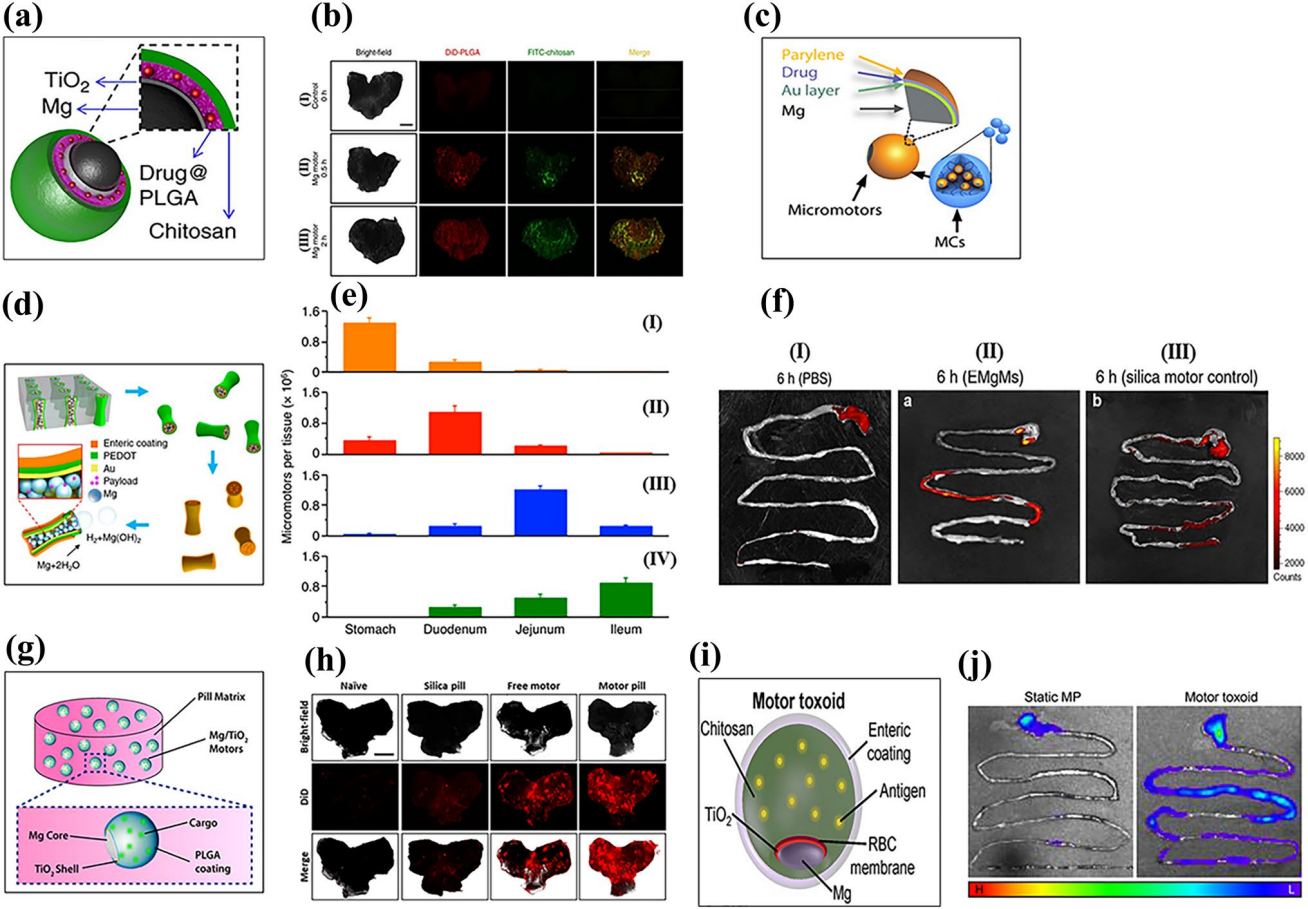
Schematic of (**a**) Mg-TiO_2_-drug@PLGA-chitosan micromotor. Reproduced with permission [144]. Copyright 2017, Springer Nature. (**b**) Bright-field and fluorescence images of the luminal lining of the mouse stomach: (I) control, (II) Mg micromotor after 30 min and (III) 2 h of oral administration. Reproduced with permission [144]. Copyright 2017, Springer Nature. (**c**) PACT-controlled drug-loaded microcapsule. Reproduced with permission [143]. Copyright 2019, The American Association for the Advancement of Science. (**d**) Fabrication of EMgMs. Reproduced with permission [195]. Copyright 2016, American Chemical Society. (**e**) ICP analysis of micromotors retention in the GI tract: (I) bare Mg, EMgMs with (II) thin, (III) medium and (IV) thick polymer coating. Reproduced with permission [195]. Copyright 2016, American Chemical Society. (**f**) Fluorescence images of mouse GI tract (I) control, (II) EMgMs coated with medium polymer and loaded with rhodamine 6G, and (III) silica motor control after 6 h of oral administration. Reproduced with permission [195]. Copyright 2016, American Chemical Society. (**g**) Schematic of a microrobot pill containing an Mg/TiO_2_/PLGA micromotor in a lactose/maltose pill matrix. Reproduced with permission [142]*.* Copyright 2018, American Chemical Society. (**h**) Bright-field fluorescence image (DiD dye loaded microrobot) of the luminal lining of the mouse stomach: (I) naïve, (II) silica pill (III) free Mg micromotor, and (IV) Mg micromotor pill at 4 h after oral administration. Reproduced with permission [142]*.* Copyright 2018, American Chemical Society. (**i**) Mg/TiO_2_/RBC-toxin/chitosan/enteric-coated motor toxoid. Reproduced with permission [196]. Copyright 2019, American Chemical Society. (**j**) Representative image of the mouse GI tract: (I) static micromotor (DiD labeled) and (II) motor toxoids at 6 h after oral gavage. (H, high fluorescence; L, low fluorescence). Reproduced with permission [196]. Copyright 2019, American Chemical Society

As an alternative approach, Avila et al. fabricated Mg-based micromotors loaded with the antibiotic, clarithromycin (CLR) [144]. The Mg microsphere was asymmetrically coated with a thin, uniform layer of TiO_2_ and then coated layer-by-layer with drug-loaded PLGA and chitosan (Fig. 5a). The micromotors attached to the mucosal layer of the stomach wall by their outer chitosan layer. The drug-loaded Mg micromotors had an average speed and lifetime of 120 µm s^−1^ and 6 min, respectively, in simulated gastric fluid (pH 1.3). The lifetime could be tuned by controlling the size of the opening. Efficient propulsion of CLR-loaded Mg micromotors is required to attach to the stomach wall and achieve a therapeutic effect. Furthermore, the continuous degradation of Mg reduces the concentration of H^+^ ions in gastric fluid and neutralizes its pH. In vivo mouse studies showed that CLR-loaded Mg-based micromotors had better retention and interactions with stomach tissue (Fig. 5b). This significantly reduced bacterial infection compared to passive drug delivery.

Although chemically propelled Mg micromotors show promise for the treatment of GI tract diseases due to their bubble recoil-based autonomous propulsion, imaging and precise control of these micromotors for sustainable drug release remain challenges to be overcome. To this end, Wu et al. developed a photoacoustic computed tomography (PACT) guided Mg-based micromotors that exhibited controlled propulsion and long-term in vivo retention [143]. Micromotor capsules (MCs) were fabricated in two steps. In the first step, spherical Mg particles were coated with Au, an alginate-drug solution, and a perylene layer as a shell scaffold. Next, complete encapsulation of the fabricated micromotors was performed using enteric gelatin (Fig. 5c). The Au layer enhanced optical absorption for PACT imaging and efficient propulsion due to galvanic corrosion. The MCs were administered to mice orally with water and evaluated by PACT imaging after anesthesia. Non-invasive PACT imaging enabled real-time monitoring of the micromotors in deep tissue (7 cm) with high resolution. Furthermore, continuous-wave near-infrared (CW-NIR) irradiation at the target site led to a phase change in the capsule material, which triggered the micromotor’s chemical propulsion. As a result, the micromotors adhered to the intestine walls, thereby prolonging their retention.

Wang et al. developed an enteric-coated Mg-based micromotor that exhibited potential for the treatment of GI tract-related bacterial diseases [195]. The enteric coating preserved the integrity of the micromotors in an acidic medium while being degraded in intestinal fluid (pH 6–7), leading to efficient propulsion. To accomplish this, researchers loaded Mg particles into an enteric-coated PEDOT/Au microtube (Fig. 5d). Due to the chemical reaction between the Mg particles and the intestinal fluid, the micromotors achieved an average speed of 60 µm s^−1^. Controlling the thickness of the enteric coating enabled selective positioning and activation of the micromotor in the GI tract. Micromotors with a thick polymer coating (1.2 µm) had an activation time of 2 h in intestinal fluid, indicating that the micromotors selectively reached the lower GI tract. An in vivo mouse model indicated that the distribution and retention of enteric-coated Mg micromotors (EMgMs) in the GI tract largely depended on the thickness of the enteric coating (Fig. 5e). Furthermore, EMgMs coated with a medium polymer exhibited higher fluorescence intensity than silica microsphere-loaded PEDOT/Au microtubes, which could be attributed to the enhanced retention of EMgMs in the GI tract as a result of propulsion and collision with the porous slimy mucus layer (Fig. 5f).

In a separate study, Wang et al. fabricated a swallowable Mg micromotor pill and evaluated its in vivo response in a gastric environment [142]. Asymmetric Janus Mg micromotors coated with TiO_2_ and PLGA were embedded in a pill matrix of inactive excipients (lactose/maltose) (Fig. 5g). The dissolution of the pill matrix in gastric fluid led to the release of Mg micromotors, which were autonomously propelled by the hydrogen bubble recoil mechanism until they were completely dissolved. Pill matrix dissolution was size and temperature dependent, with larger pills (5 × 3 mm^2^) dissolving more rapidly at 37 °C (6 min) than at 22 °C (8 min). Also, the excipient matrix did not impact the propulsion behavior of the released Mg micromotors. To understand the in vivo retention of the micromotors in stomach tissue, the researchers used fluorescently labelled (DiD) micromotors. The results showed that pills with Mg/TiO_2_/PLGA@DiD/chitosan micromotors had a higher fluorescence intensity than free micromotors (Fig. 5h).

The efficacy of orally administrated vaccines needs to be enhanced [196]. To this end, Wang’s group has developed Mg-based biomimetic micromotors for oral vaccine delivery. Using the cell membrane-coating technique, they immobilized and neutralized model bacterial toxins on the surface of micromotors. The Mg microspheres were asymmetrically coated layer-by-layer with TiO_2_, toxin (staphylococcal α-toxin) embedded in a red blood cell (RBC) membrane and mucoadhesive chitosan, and encapsulated with a pH-responsive enteric polymer coating for selective activation in the GI tract (Fig. 5i). In simulated gastric fluid, bare Mg/TiO_2_ and motor toxoids displayed almost identical propulsion speeds (200 µm s^−1^). However, for motor toxoids, a speed reduction (24%) was observed in intestinal fluid at neutral pH due to the integrity of the membrane and chitosan layer, which might hinder fuel access to the motor core. A favorable cell-motor interaction was observed for motor toxoids compared to static micromotors, as evidenced by the significant increase in membrane fragment uptake. Overall, the enteric-coated motor toxoid exhibited no cytotoxicity up to 2 mg/mL. In vivo studies also indicated enhanced retention of motor toxoids and an enhanced immune response against α-toxins in orally vaccinated mice (Fig. 5j).

Chen et al. reported self-destroyed transient material-based biocompatible micromotors for the treatment of GI tract diseases [140]. Mg/ZnO Janus micromotors exhibited complete degradation after 18 min in 0.5 M NaHCO_3_. Although the entire Mg core did not degrade, Mg/ZnO micromotors were observed to move at a velocity of 24.7 µm s^−1^ for only 1 min, possibly because of their less reactive surface area, which hampers bubble nucleation and growth. The authors also designed Mg/Si Janus micromotors that showed complete degradation in 5 h. The prolonged degradation time of the outer Si shell could be useful for drug delivery platforms, bioimaging, or biosensing.

Maric et al. developed a self-propelled pH-responsive Mg micromotor for drug delivery in the GI tract [146]. Mg/Au Janus micromotors were fabricated and loaded with the drug furosemide, and a pH-responsive enteric coating was applied to protect the drug. Drug- and polymer-coated Janus Mg micromotors showed significantly enhanced lifetimes and velocities compared to bare Mg/Au micromotors (Table 2). The motion of the majority of the micromotors was linear, suggesting that hydrodynamic drag and the propulsion force were in the same direction. In vitro drug-release studies indicated that the micromotor fully released the drug within 3 min.

**Table 2:** Process and performance parameters of a magnesium-based micromotors for biomedical applications

Sl. no	Composition	Particle size (µm)	Deposition parameters	Velocity (µm/s)	Carrier	Drug used	Actuation method	Average life time (s)	Immersion medium	In vivo model	Refs
*GI tract applications*											
1	Mg/TiO_2_/Drug@PLGA/chitosan	20 ± 5	TiO_2_ deposition using ALD at 100 °C for 120 cycles	*V*_avg_ = 120	None	CLR	Chemical	180	0.1 N HCl	Male mice	[144]
2	Mg/Au/drug@alginate/parylene C	20 ± 5	Au deposition (100 nm) through E-beam evaporator	*V*_avg_ = 45 (PBS) *V*_avg_ = 43 (intestinal fluid)	Enteric-coated microcapsule	DOX	NIR-activated propulsion. PACT used for real time imaging	–	PBS/intestinal fluid	Nude mice	[143]
3	PEDOT/Au microtube loaded with Mg particles	5	Electroplating technique	*V*_avg_ = 60	Enteric-coated microtube	–	Chemical	60	Gastric/intestinal fluid	Male mice	[195]
4	Mg/TiO_2_/cargo@PLGA	20 ± 5	TiO_2_ deposition using ALD at 100° C for 3,000 cycles	_˜_260 (22° C) _˜_300 (37° C)	Lactose (60%)/maltose (40%) pill		Chemical	300 (Pill dissolution time)	Simulated gastric fluid	Male CD-1 mice	[142]
5	Mg/TiO_2_/RBC-toxin/chitosan	20 ± 5	TiO_2_ deposition using ALD at 100 °C for 3,000 cycles	200 (gastric fluid) 152 (intestinal fluid)	Enteric coating	Staphylococcal α-toxin	Chemical	–	Gastric/intestinal fluid	Male CD-1 mice	[196]
6	Mg/ZnO	30	ZnO shell thickness 100 nm	24.7	–	–	Chemical	60	0.5 M NaHCO_3_	–	[140]
7	Mg/Au/drug/Eudragit® L100	20 ± 5	Au shell thickness 40 nm	46.5	–	Furosemide	Chemical	250	3 M NaCl and 0.5 wt% Triton X-100	–	[146]
*ROS scavenging*											
1	Mg/drug@PLGA	25	–	57 ± 19	–	DOX	Chemical	270	SBF	–	[147]
2	Mg/HA hydrogel/PLGA	16	Thickness of hydrogel and PLGA 2.5 µm	40.1 (SSF) 45.5 (PBS)	–	Hyaluronic Acid	Chemical		Simulated synovial fluid/PBS	CIA Rats	[208]
3	Mg@mesoporous Silica	20	SiO_2_ shell thickness 100 nm	–	–	–	Chemical	22 h	SBF	–	[209]
*Imaging and other applications*											
1	Mg-Ni	25	Ni layer 100 nm	301.7 (at 10 Hz and 24 µm particle size)	–	–	Chemical + magnetic	76	aqueous solution containing HCO_3_^−^, H^+^ and Cl^−^	–	[212]
2	Mg/TiO_2_/PLL/MΦ	20–25/10–15	TiO_2_ deposition using ALD at 100° C for 3,000 cycles	190.5 ± 13.24/62.8 ± 4.3	–	–	Chemical	300	Simulated gastric fluid	–	[215]
3	Mg/AuNP@alginate/RBC	20 ± 5	Incubation with chitosan-stabilized Au, ALG and RBC separately	172			Chemical	> 2	0.08 M NaCl s	–	[214]

#### Reactive Oxygen Species Scavenging

Excessive generation of hydroxyl radical (^·^OH), a cytotoxic reactive oxygen species (ROS), in mitochondria can lead to oxidative stress. Major diseases, including cancer, diabetes, Alzheimer, and Parkinson disease, are associated with elevated levels of ROS in tissues [197–201]. Minimizing the ROS levels in cancerous cells leads to apoptosis, making ROS scavenging a potential therapeutic strategy for diseases related to elevated ROS levels.

Hydrogen is a potent antioxidant with therapeutic potential. Hydrogen therapy scavenges ROS from tumor cells and inhibits their growth [202, 203]. Hydrogen can also balance cellular redox homeostasis and selectively reduce toxic oxidative radicals, such as hydroxyl radicals. In addition, hydrogen activates endogenous antioxidant enzymes and downregulates proinflammatory cytokines [202, 203]. However, the therapeutic potential of hydrogen is limited by its low solubility in physiological solutions (0.8 mM) [204]. Therefore, hydrogen carriers—such as palladium hydride (PdH_0.2_) nanocrystals, magnesium bromide (MgB_2_) nanosheets, and iron nanoparticles (Fe NPs)—have been developed to enhance therapeutic efficacy and enable targeted and controlled delivery of molecular hydrogen [205, 206]. However, the above approaches use non-degradable porous micro- or nanoparticles, which have limited loading capacity and tissue penetration, and toxicity concerns.

**Fig. 6:**
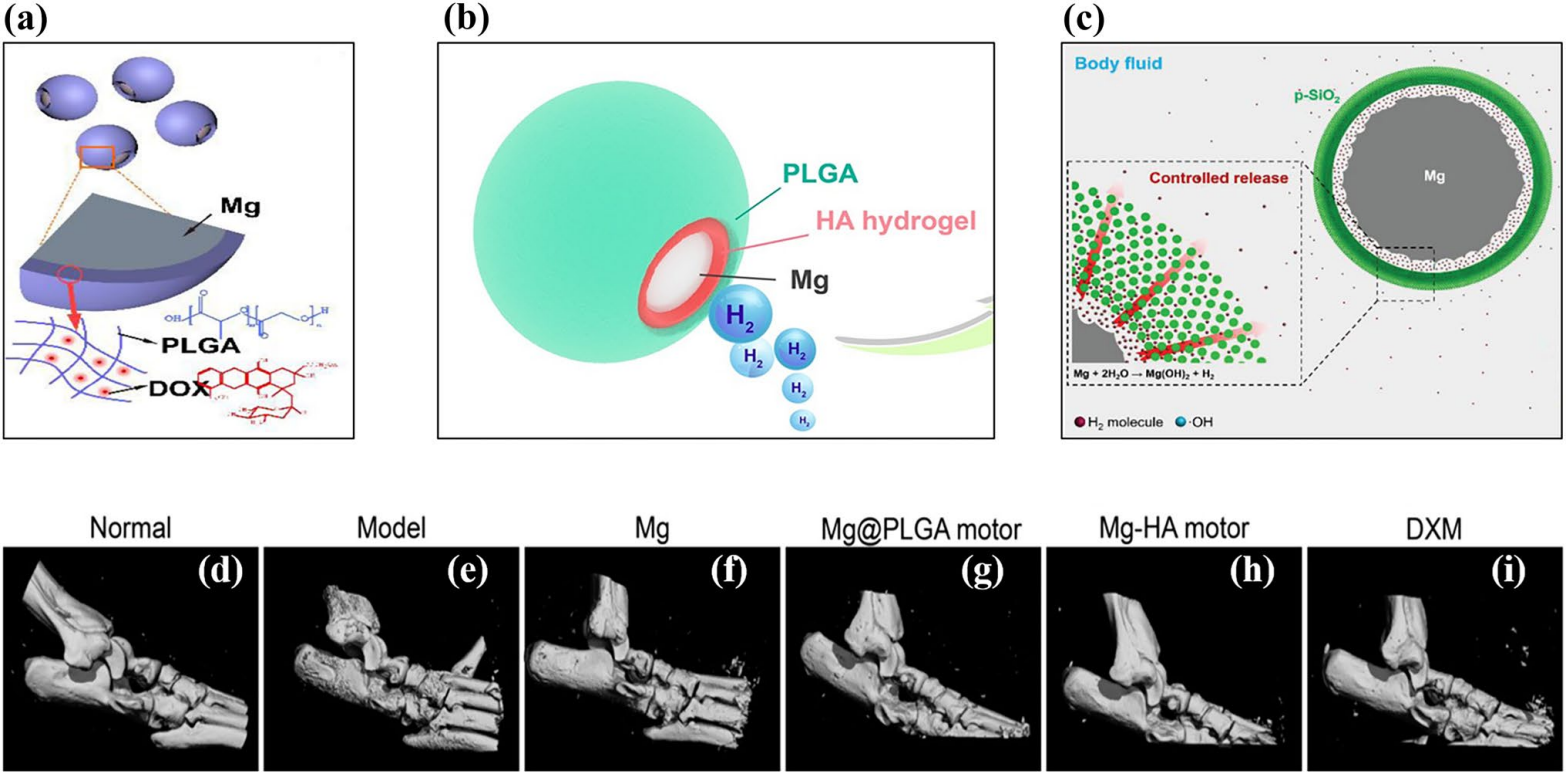
Schematic representation of (**a**) Mg-PLGA-DOX micromotor. Reproduced with permission [147]. Copyright 2020, Elsevier. (**b**) Mg-HA-PLGA micromotor. Reproduced with permission [208]. Copyright 2021, American Chemical Society. (**c**) Mesoporous SiO_2_-coated Mg micromotor. Reproduced with permission [209]. Copyright 2018, Wiley Publication. (**d**)–(**i**) Representative micro-CT analysis of rat ankle joint after 29 days of treatment. Reproduced with permission [208]. Copyright 2021, American Chemical Society

Magnesium-based Janus micromotors were developed for long-term hydrogen therapy. Liu et al. synthesized doxorubicin (DOX)-loaded PLGA-coated, Mg-based micromotors for synergistic hydrogen chemotherapy (Fig. 6a) [147]. The micromotor exhibited an average speed of 57 ± 19 μm s^−1^ with a lifetime of 4.5 min. The Janus-type Mg micromotor group showed a 12-fold increase in ROS scavenging efficacy compared to the non-micromotor group due hydrogen-mediated disruption of redox homeostasis.

Rheumatoid arthritis (RA) is a chronic disease that causes bone and cartilage destruction. The degradation of hyaluronic acid in the synovial joints is closely related to cartilage damage [207]. Although the cause of RA is unclear, excessive ROS production is associated with its development. Xu et al. developed Mg-based micromotors as hydrogen generators to reduce the ROS level in RA patients (Fig. 6b) [208]. The asymmetrically coated Mg-hyaluronic acid (HA) micromotor was deposited with biodegradable PLGA for the controlled release of hydrogen. The coating of HA and PLGA did not affect the motion of the Mg-based micromotors but prolonged their lifetime to approximately 5 min. Active motion of micromotors could be detected by ultrasound imaging using the hydrogen bubble due to the acoustic impedance difference. The prepared microparticles did not show any toxic response, even at a higher concentration (2 mg mL^−1^). Furthermore, the in vitro response of the micromotors reduced the overproduction of proinflammatory cytokines (TNF-α, IL-1β, and IL-6), which is considered to benefit RA. In a rat model of collagen-induced arthritis, disease progression was reduced by the synergistic effect of hydrogen gas delivery and HA (Fig. 6d–i).

Kong et al. synthesized core–shell type Mg with mesoporous SiO_2_ for hydrogen therapy (Fig. 6c) [209]. In this design, the porous structure of the mesoporous silica shell allows the physiological solution to react with the Mg core. The controlled generation and release of hydrogen were achieved by the barrier effect of the mesoporous silica shell, and the lifetime of the Mg micromotor was positively correlated with the thickness of the mesoporous silica layer due to an increase in the diffusion path of the reactant. A mesoporous SiO_2_ shell upon bare Mg enhanced cell protection threefold compared to bare Mg.

#### Imaging and Other Applications

Ultrasound imaging is low cost, non-invasive, allows real-time monitoring, and does not require radiation exposure. Typically, microbubbles are employed as contrast agents to improve acoustic contrast in target tissues and surrounding areas [210, 211]. However, the complex manufacturing and storage of microbubbles can limit image quality. To address this issue, Feng et al. developed Janus-type Mg/Ni micromotors that react with body fluids, releasing microbubbles as an ultrasound contrast agent to obtain high-quality images [212]. The micromotor velocity increased with ion concentration because of a higher rate of passivation layer removal of Mg(OH)_2_. Magnetic navigation enabled micromotors to move from neutral to acidic pH regions due to the presence of Ni in their structure. Under acidic conditions, the micromotor generated a large number of bubbles, which enabled precise imaging of the cavity organ. Targeted lesion imaging was achieved by combining ultrasound and a magnetic field. The echogenicity between the target tissue and microbubbles generated from Mg/Ni micromotors provided a clear ultrasound image. In vitro biocompatibility analysis using the tetrazolium-based colorimetric 3-(4,5-dimethylthiazol-2-yl)-2,5-diphenyltetrazolium bromide (MTT) assay did not reveal a cytotoxic response to the micromotors.

Zhou et al. investigated the photothermal and anti-tumor effects of biodegradable Mg/PLGA micro- and nanorobots [213]. Spherical micro- and nanomotors were developed by microfluidic fabrication, and their efficacy for photothermal therapy was evaluated. The performance of photothermal therapy depended on the Mg content, not carrier (PLGA) thickness. In vivo tumor models demonstrated remarkable tumor shrinkage in animals treated with Mg/PLGA microspheres under NIR light.

A cell-mimicking water-powered Mg-based Janus micromotor asymmetrically coated with RBCs, Au NPs, and alginate was developed [214]. The RBC-coated micromotors efficiently attracted, captured, and neutralized toxins in biological fluids. RBC-Mg micromotors efficiently cleaned α-toxin-containing biological solutions due to their propulsion. The addition of negatively charged iron oxide NPs to the micromotor structure enhanced control of their movement.

Wang et al. reported Mg micromotors with living macrophages (MΦ) in their extreme outer layers [215]. Initially, the particles were coated with a TiO_2_ and poly (L-lysine) (PLL) layer, to which MΦ attached via electrostatic interactions. The cell membrane of gram-negative bacteria releases lipopolysaccharide (LPS), which is responsible for sepsis. MΦ can bind to LPS and remove it from the body. The MΦ-Mg micromotor removed LPS (66.82 ± 6.31%) more efficiently than free MΦ (53.34 ± 4.48%). Bubble propulsion resulted in the rapid movement (127.3 µm s^−1^) of MΦ-Mg micromotors, enabling them to bind more LPS Mg-based micromotors for biomedical applications (summarized in Table 2).

Xiong et al. first proposed a stimuli-induced “hovering” strategy for self-propelled Mg micromotors with motion responsive to both environmental temperature and H_2_O_2_ concentration [216]. The noble metal Platinum (Pt) and temperature-sensitive poly(N-isopropylacrylamide) (PNIPAM) were deposited asymmetrically on Mg micromotor in sequence. The Mg-based micromotor showed hydrogen bubble propulsion in an aqueous solution of NaHCO_3_ and poly(vinylpyrrolidone) (PVP) due to a self-consuming Mg-H_2_O reaction. The propulsion mechanism transformed from the self-consuming Mg-H_2_O reaction to Pt-catalyzed H_2_O_2_ decomposition because of the presence of H_2_O_2_ in the surrounding environment. The change in the propulsion mechanisms would extend lifetime of the micromotor. Furthermore, the solution temperature also plays an important role in achieving different propulsion mechanisms. PNIPAM hydrogel has a large diffusion constant within the solution, below its lower critical solution temperature (LCST/32 °C). Consequently, the PNIPAM layer readily allows the H_2_O_2_ aqueous solution to permeate, initiating a reaction with Pt. This reaction triggers temperature-induced hovering motion due to the formation of oxygen (O_2_) bubbles on both sides. The velocity of Mg micromotors increased from 14.3 to 28.7 μm s^−1^ during their transition from an aqueous NaHCO_3_ solution to a 0.1 wt% H_2_O_2_ solution at 38 °C. The enhanced velocity could be attributed to the higher production of O_2_ bubbles, which stems from both the higher rate constant of Pt-catalyzed H_2_O_2_ decomposition and the lower critical nucleation concentration of O_2_.

Kong et al. developed Mg/Pt Janus micromotor to sense glucose level in human serum without any additional toxic fuels or surfactants [153]. The sensing device is a screen printed with a three-electrode system in which the working electrode was modified with glucose oxidase (GO_x_) and glutaraldehyde (GTA). The system follows the principle of second-generation glucose biosensor. In this system, ferrocenemethanol (FcMeOH) enhances the heterogeneous electron transfer process when glucose is broken down enzymatically by GO_x_. The electrochemical conversion of FcMeOH into Fc^+^ MeOH indicates the glucose concentration within the solution, while generating an electric signal. The developed Mg/Pt micromotor exhibited fast motion and vigorous bubbling in the solution, leading to an increased flow of molecules and ions. Consequently, the oxidation of glucose and FcMeOH on the electrode surface elevated the detection sensitivity. The presence of glucose in the solution had no impact on the average velocity and lifetime of the micromotor. The enhanced signal current for glucose in human serum when using Mg/Pt Janus micromotors could be attributed to the improved mass transfer within the solution due to the rapid motion of the micromotors. Furthermore, the chronoamperometric data corroborated the significant role of Mg/Pt micromotors in enhancing the current response at millimolar glucose concentration.

### Environmental Applications

Conventional water treatment methods typically rely on oxidizing agents, which can generate harmful by-products that pose health risks. Researchers have developed Mg-based micromotors as a promising, environmentally friendly technology capable of removing environmental pollutants by enhanced localized fluid mixing. The two primary mechanisms contributing to this enhanced mixing are microbubble generation and fluid flow induced by motion. Mg-based Janus micromotors do not need fuel and produce no harmful chemicals during clean-up. Therefore, researchers have sought to incorporate various functional groups or reactive surfaces onto Mg Janus micromotors to enhance their potential for environmental remediation.

**Fig. 7:**
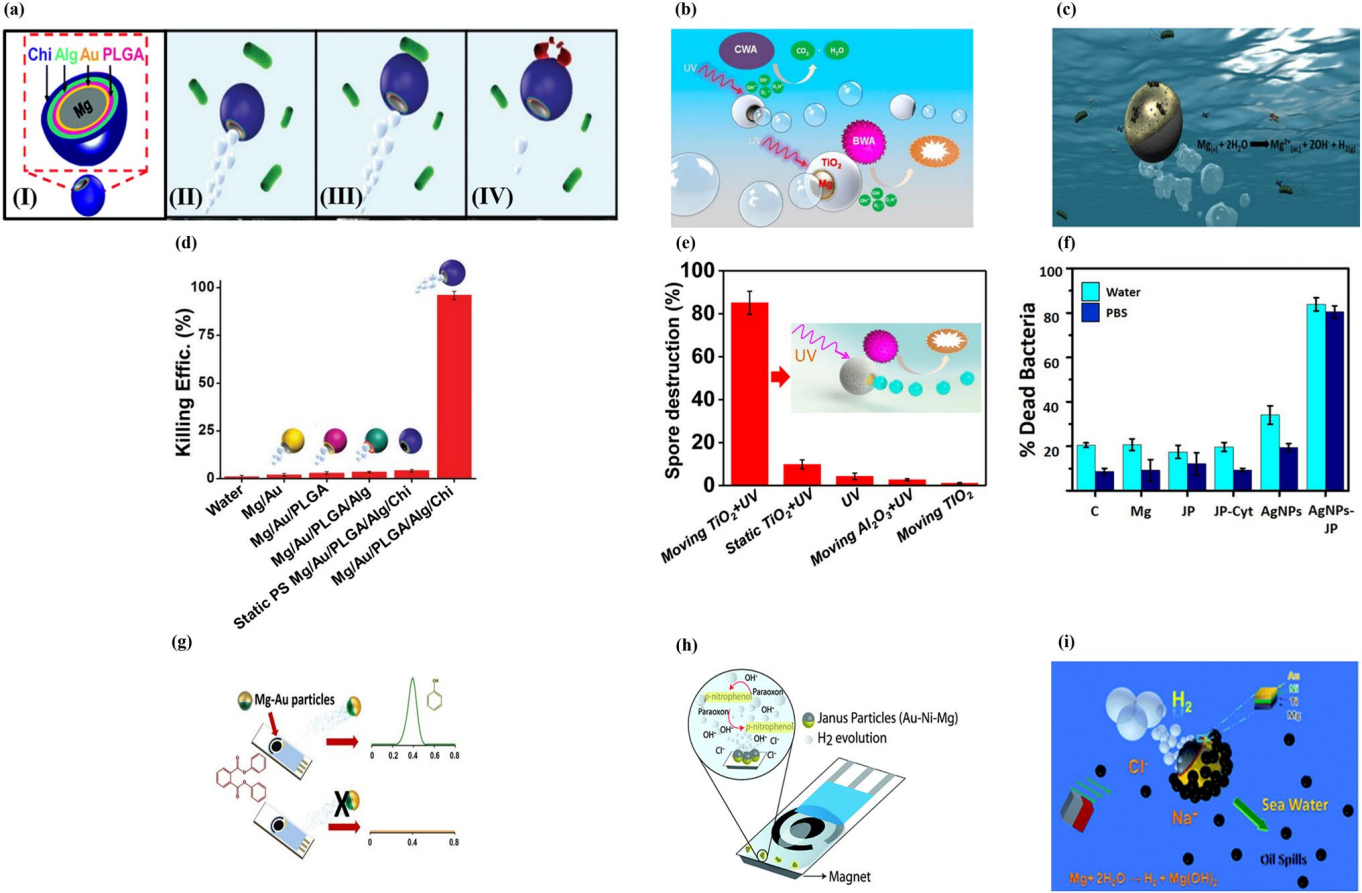
(**a**) (I) Structure of Mg/Au/PLGA/Alg/Chi micromotors, (II) water-powered Mg/Au/PLGA/Alg/Chi micromotor approaching bacteria, (III) contact between micromotor and *E*. *coli*, (IV) micromotor killing bacteria. Reproduced with permission [220]. Copyright 2017, Royal Society of Chemistry. (**b**) Self-propulsion and photocatalytic degradation of biological and chemical warfare agents by a chemically propelled TiO_2_/Au/Mg micromotor. Reproduced with permission [96]. Copyright 2014, American Chemical Society. (**c**) Possible mechanism of the bactericidal activity of the water-propelled, AgNP-coated Janus micromotor. Reproduced with permission [150]. Copyright 2017, American Chemical Society. Statistical plot of *E. coli* killing by (**d**) Mg/Au/PLGA/Alg/Chi. Reproduced with permission [220], Copyright 2017, Royal Society of Chemistry. Spore destruction efficiency of (**e**) TiO_2_/Au/Mg. Reproduced with permission [96]. Copyright 2014, American Chemical Society. (**f**) Percentage of dead *E. coli* in contact with 0.5 mg Mg, Janus microparticles (JPs), cysteamine-modified JPs (JP-cyt), and AgNP-coated microrobots at pH 6. Reproduced with permission [150]. Copyright 2017, American Chemical Society. (**g**) Mg/Au Janus micromotor-based strategy for the simultaneous degradation/detection of DPP. Reproduced with permission [221]. Copyright 2016, American Chemical Society. (**h**) Mechanism of detection of organophosphorus nerve agents by Mg micromotors. Reproduced with permission [222]. Copyright 2015, Royal Society of Chemistry. (**i**) Seawater-driven magnetically guided alkanethiol-modified Mg micromotor for environmental oil remediation. Reproduced with permission [50]. Copyright 2013, Royal Society of Chemistry

Chitosan has broad-spectrum antimicrobial activity against various bacteria and fungi [217–219]. The electrostatic interaction between chitosan and the cell membrane is responsible for its antibacterial properties. Delezuk et al. developed self-propelled Mg/Au/PLGA/Alg/chitosan-coated micromotors that could swim through bacteria-contaminated water [220]. The outer chitosan layer mechanically damages bacterial cells upon contact, resulting in highly efficient bacterial killing (Fig. 7a). Macro-galvanic corrosion between the Mg and Au layers is responsible for the highly efficient bubble propulsion of the Mg-chitosan micromotors. Notably, the Mg-Chitosan micromotor demonstrated a bacterial killing efficiency of > 96% in drinking water within 10 min due to its efficient propulsion and chitosan’s antibacterial properties (Fig. 7d). This antibacterial micromotor showed a bacterial killing efficiency of > 90% in seawater and freshwater.

The photocatalytic decomposition of chemical and biological warfare agents (CBWAs) using self-propelled TiO_2_-coated Mg micromotors is a promising new strategy (Fig. 7b) [96]. TiO_2_/Au/Mg micromotors can rapidly and completely remove CBWAs without the need for external fuel. During the autonomous propulsion of the micromotors in the water, UV activation generates strong oxidative species on the TiO_2_ surface. Remarkably, a small quantity of micromotors (2.25 mg) degraded the nerve agent stimulant methyl paraoxon by > 90% within 10 min. Furthermore, bubble-propelled Mg/Au/TiO_2_ micromotors significantly reduced (86%) *Bacillus anthracis* spores compared to TiO_2_ and Al_2_O_3_ (Fig. 7e). The combination of autonomous propulsion and surface photocatalytic reaction accounts for the high efficiency of these micromotors in removing CBWAs from water.

Silver nanoparticles (AgNPs) were added to magnetically guided Mg micromotors for bacterial killing in a contaminated aqueous medium [150]. Mg particles were coated with Fe and Au layers by the electron beam metal evaporation method, and AgNPs were attached to the Au surface (Fig. 7c). The enhanced bacterial killing of the Mg micromotors was attributable to their autonomous propulsion, which promoted direct contact between AgNPs and bacteria, leading to selective transportation of Ag^2+^ into the cytoplasm. Most bacteria adhering to AgNP-embedded Mg/Fe/Au particles were dead. The Au layer on the Mg micromotor captured and killed the bacteria. The AgNP-loaded Mg/Fe/Au micromotor removed > 80% of bacteria from contaminated water within 15 min (Fig. 7f). The magnetic properties of the Fe layer were used to collect the micromotors together with the captured dead bacteria by magnetic guidance.

Diphenyl phthalate (DPP) is a harmful plasticizer commonly found in food packaging and serves as an indirect food additive. Rojas et al. demonstrated that Mg-based Janus-type micromotors could be used in food quality control applications [221]. Mg/Au Janus particles were used to detect DPP in food products without the use of expensive equipment (Fig. 7g). Mg/Au micromotors released hydrogen bubbles, which provided autonomous motion and enhanced mixing. Additionally, they released hydroxyl ions, which converted non-electro-active DPP into electro-active phenol, resulting in an increased analytical signal by differential pulse voltammetry (DPV).

Cinti et al. reported strip-based electrochemical measurement using Mg micromotors for the rapid degradation and detection of organophosphorus (OPH) nerve agents without the need for external devices (Fig. 7h) [222]. Degradation of Mg/Ni/Au microparticles increased the medium pH, accelerating the conversion of non-detectable paraoxon to detectable p-nitrophenol. The microbubbles generated by the micromotors promoted rapid electrochemical reactions and enhanced mixing. Also, the magnetic anchoring of the micromotors was used to restrict their movement toward the electrode area to avoid interference with the reaction. The strip-based paraoxon assay exhibited 15-fold higher sensitivity than non-engineered bare screen-printed electrodes.

Gao et al. reported ecofriendly degradable Mg micromotors with nickel-Au bilayer patches propelled by seawater (Fig. 7i) [50]. The bilayer patch integrated into the micromotor structure enabled its magnetic guidance by surface modification. The micromotors showed autonomous propulsion in seawater due to macro-galvanic corrosion and chloride pitting corrosion, with the velocity depending on the ion concentration of the medium. No bubble propulsion was observed when the micromotors were in pure water or sodium nitrate solution. However, their fast movement (300 µm s^−1^) resulted from pitting and macro-galvanic corrosion in chloride-rich solutions. Mg/Ti/Ni/Au micromotors were surface-functionalized with long-chain alkanethiols for on-the-fly removal of oil droplets. Furthermore, the Ni in the micromotors facilitated their magnetic guidance to capture and transport motor oil droplets from seawater. Table 3 summarizes Mg-based micromotors for environmental remediation.

**Table 3:** Process and performance parameters of a magnesium-based micromotors for environmental applications

Sl. no	Composition	Particle size (µm)	Thickness of deposited layer (nm)	Velocity (µm/s)	Pollutant	Antibacterial/pollutant removal agent/sensing	Actuation method	Cleaning time (s)	Immersion medium	Bacteria killing efficiency	Refs
1	Mg/Au/PLGA/Alg/chitosan	20		36.5 (drinking water) 72.6 (seawater)	E. coli	Chitosan	Chemical	600	Drinking water/seawater	96%	[220]
2	Mg/Au/TiO_2_	20	ALD for TiO_2_ deposition	80	CBWA	TiO_2_	Chemical	900	0.08 M NaCl	86%	[96]
3	Mg/Ni/Ti/Au	20	80 nm Ni/20 nm Ti/10 nm Au	90	Motor oil droplet	Alkanethiol	Chemical + magnetic	60	Seawater	–	[50]
4	Mg/Au	20 ± 5	20 nm layer of Au	108 ± 18	Diphenyl phthalate		Chemical	300	Milk	_˜_100%	[221]
5	Mg/Ni/Au	44	80 nm Ni/10 nm Au	–	Organophosphorus (OP) nerve agent p-nitrophenol	–	Chemical + magnetic	–	–	–	[222]
6	Mg/Fe/AgNP@Au	15 ± 5	–	26.9 ± 1.8 (pH 5)	*E. coli*	AgNP	Chemical + magnetic	–	Seawater	> 80%	[150]

## Zinc-Based Micromotors

Zn can be used for self-sacrificing micromotors that do not cause toxicity after degradation. Zn-based micromotors have efficient hydrogen bubble propulsion in an acidic environment because of the Zn–acid reaction. The velocity of the Zn-based micromotor could be tuned by adjusting the medium pH and or the design of the micromotor.

**Fig. 8:**
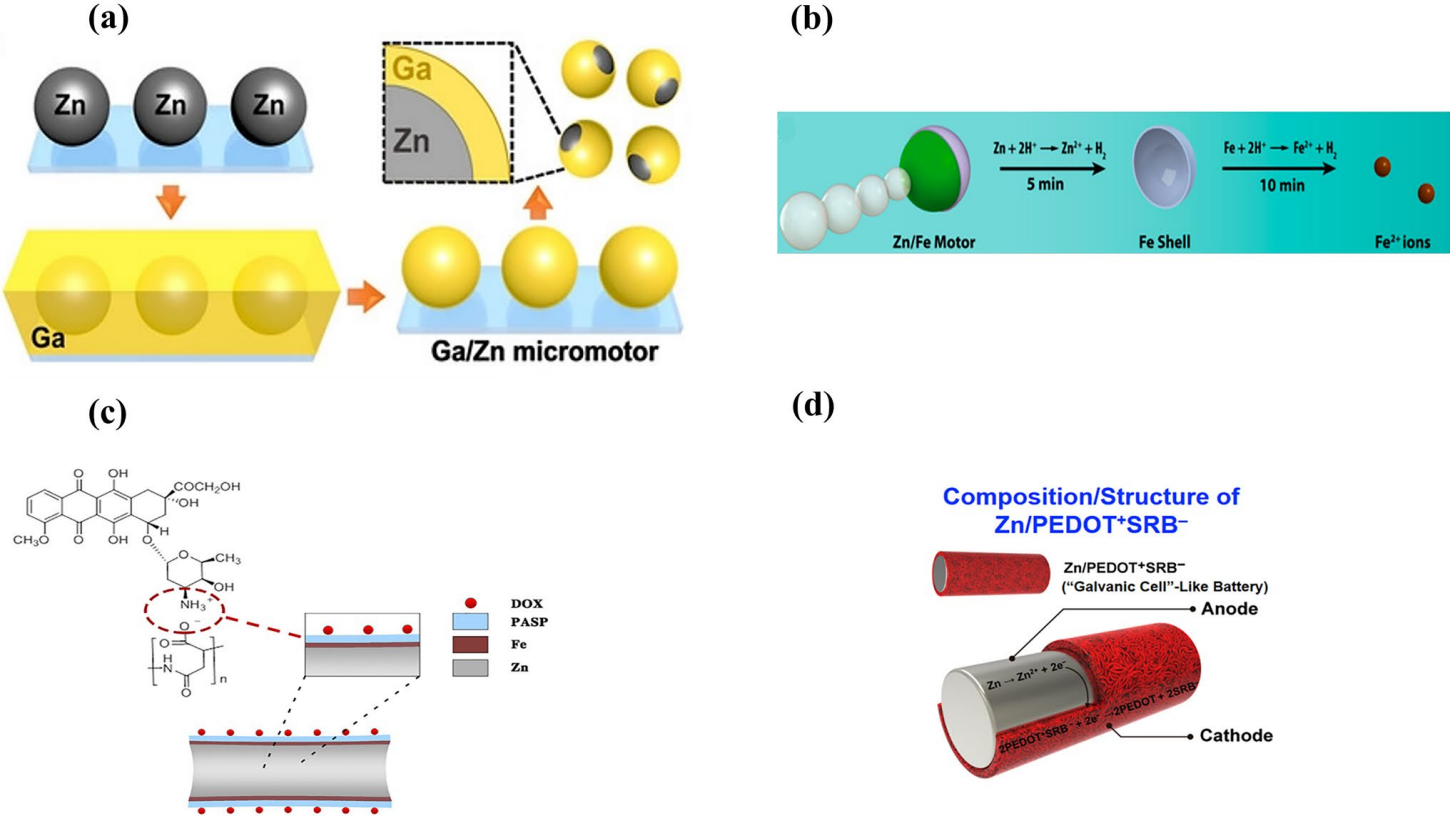
Schematic of the fabrication of a (**a**) Zn/Ga micromotor. Reproduced with permission [223]. Copyright 2021, Wiley Publication. (**b**) Zn/Fe Janus micromotor in simulated gastric acid. After Zn degradation, the Fe shell degrades to Fe^2+^ ions. Reproduced with permission [140]. Copyright 2016, American Chemical Society. (**c**) DOX/PASP/Fe-Zn micromotor. Reproduced with permission [224]. Copyright 2019, American Chemical Society. (**d**) Zn/PEDOT + SRB − microrod. Reproduced with permission [226]. Copyright 2019, Elsevier

Lin et al. reported a gallium/zinc (Ga/Zn) micromotor for targeted antibacterial chemotherapy [223]. The Ga/Zn micromotor was fabricated by partially coating a Ga layer on a Zn core by microcontact printing (Fig. 8a). These hydrogen-powered micromotors exhibited rapid movement (382.3 µm s^−1^) because of the galvanic effect of Ga and Zn in gastroenteric acid. Complete degradation of Ga/Zn micromotors in gastroenteric acid led to the release of Ga^III^ ions, which showed antibacterial activity against *H. pylori*. The movement of Ga/Zn micromotors changes the diffusion pattern of Ga from passive to active, which explains their enhanced antibacterial efficacy.

Chen et al. developed Zn/Fe Janus micromotors and evaluated their performance in simulated gastric acid [140]. The micromotors reached a velocity of 51.2 µm s^−1^ and showed active movement for 2 min in gastric acid. After 5 min of immersion, a semi-opaque Fe shell was found in the acidic solution, suggesting complete degradation of the Zn core (Fig. 8b). Inductive coupled plasma (ICP) analysis of the acidic medium indicated a significant increase in the Fe ion concentration after 10 min of immersion. This could be attributed to the self-destruction of the micromotors.

Wang and coworkers fabricated a Zn-core-based Fe and poly(aspartic acid) (PASP) layer containing a DOX-loaded micro-rocket to treat gastric cancer (Fig. 8c) [224]. The drug-loaded micro-rocket used gastric acid as a fuel for propulsion, and its location could be controlled magnetically. It could permeate the gastric mucus gel layer and release DOX in the stomach wall in an acidic environment. The micro-rocket moved at an average velocity of 34.0 ± 7.2 μm s^−1^ by hydrogen bubble propulsion, whereas magnetic actuation induced an average velocity of 29.2 ± 7.9 μm s^−1^. The slow velocity of magnetically controlled micro-rockets compared to self-propelled micro-rockets indicated a change in direction due to magnetic navigation. In vitro study demonstrated that a combination of self-propulsion and magnetic navigation improved drug delivery compared to an only self-propelled micro-rocket. Furthermore, in vivo drug delivery in the mouse stomach showed good agreement with the in vitro results.

PEDOT/Zn micromotor were fabricated by template-directed electrodeposition [225]. The micromotors were orally administered to the mouse stomach, and their autonomous motion, toxicity, and retention on the stomach wall were evaluated. In gastric fluid, PEDOT/Zn micromotors moved at a velocity of 60 µm s^−1^. In the highly acidic gastric environment (pH 2), the self-propelled Zn micrormotors showed propulsion for 10 min. In vivo studies indicated that Zn-based conical micromotors had enhanced penetration and retention in porous gel-like mucus layers as a result of their autonomous propulsion. Although these micromotors penetrated the gel-like mucus layer of the stomach, they did not damage epithelial cells.

Cui et al. proposed a battery-driven drug delivery system [226] fabricated by template-directed electrodeposition. It consisted of a Zn microrod and a positively charged PEDOT shell containing anionic model drug sulforhodamine B (SRB^−^) (Fig. 8d). When the device was immersed in a physiological solution, two redox reactions occurred simultaneously. At a low pH (gastric fluid), the Zn core oxidized due to its reaction with gastric fluid and moved by hydrogen bubble propulsion. By contrast, the PEDOT^+^ shell was reduced as a result of the consumption of electrons generated by Zn oxidation and the incorporated SRB^−^ drug was released. Both of these reactions were governed by the local pH. Therefore, at neutral pH localized drug release could occur around the gastric mucus layer and subcutaneous tissue. Mice treated with Zn-microrods showed more intense fluorescence (8.0 ± 1.4 × 10^8^ p s^−1^) than the Cu microrod group (2.1 ± 1.2 × 10^8^ p s^−1^). This result could be attributed to the rapid movement of Zn microrods in gastric fluid. Similarly, a significant increase in fluorescence intensity (2.6 ± 1.2 × 10^9^ p s^−1^) was noted for subcutaneously injected Zn microrods because of payload release at pH 7.4. Furthermore, a histological study did not indicate significant inflammation of stomach or subcutaneous tissue. Therefore, Zn-based devices could be used safely for drug delivery. The fabrication methods and performance parameters of Zn-based micromotors are listed in Table 4.

**Table 4:** Process and performance parameters of a zinc-based micromotors for biomedical applications

Sl. no	Composition	Particle size (µm)	Thickness of deposited layer (nm)	Velocity (µm/s)	Carrier	Drug used	Actuation method	Average life time (s)	Immersion medium	In vivo model	Refs
1	Zn/Ga	8.5 ± 0.8	0.2 µm	382.3 (pH 0.5) 161.2 (pH 2)	–	–	Chemical	33 (pH 0.5) 312 (pH 2)	Simulated gastric fluid	–	[223]
2	Zn/Fe	30	150	40	–	–	Chemical + magnetic	120	Simulated Gastric fluid	–	[140]
3	Zn/Fe/DOX@PASP		PASP layer 0.5–1 µm	34.0 ± 7.2	–	DOX	Chemical + magnetic	135 ± 37	Simulated gastric fluid	Mice	[224]
4	PEDOT/Zn		20 µm (L) 5 µm (diameter)	60	PEDOT	–	Chemical	600	Simulated gastric fluid	Mice	[225]

## Iron-Based Microrobots

Iron is the most abundant material in the Earth’s crust and an essential component of hemoglobin and myoglobin. The potential of Fe-based materials for degradable stents has been evaluated [227–229]. Fe can also be used in microrobots based on its favorable biocompatibility and magnetic response.

**Fig. 9:**
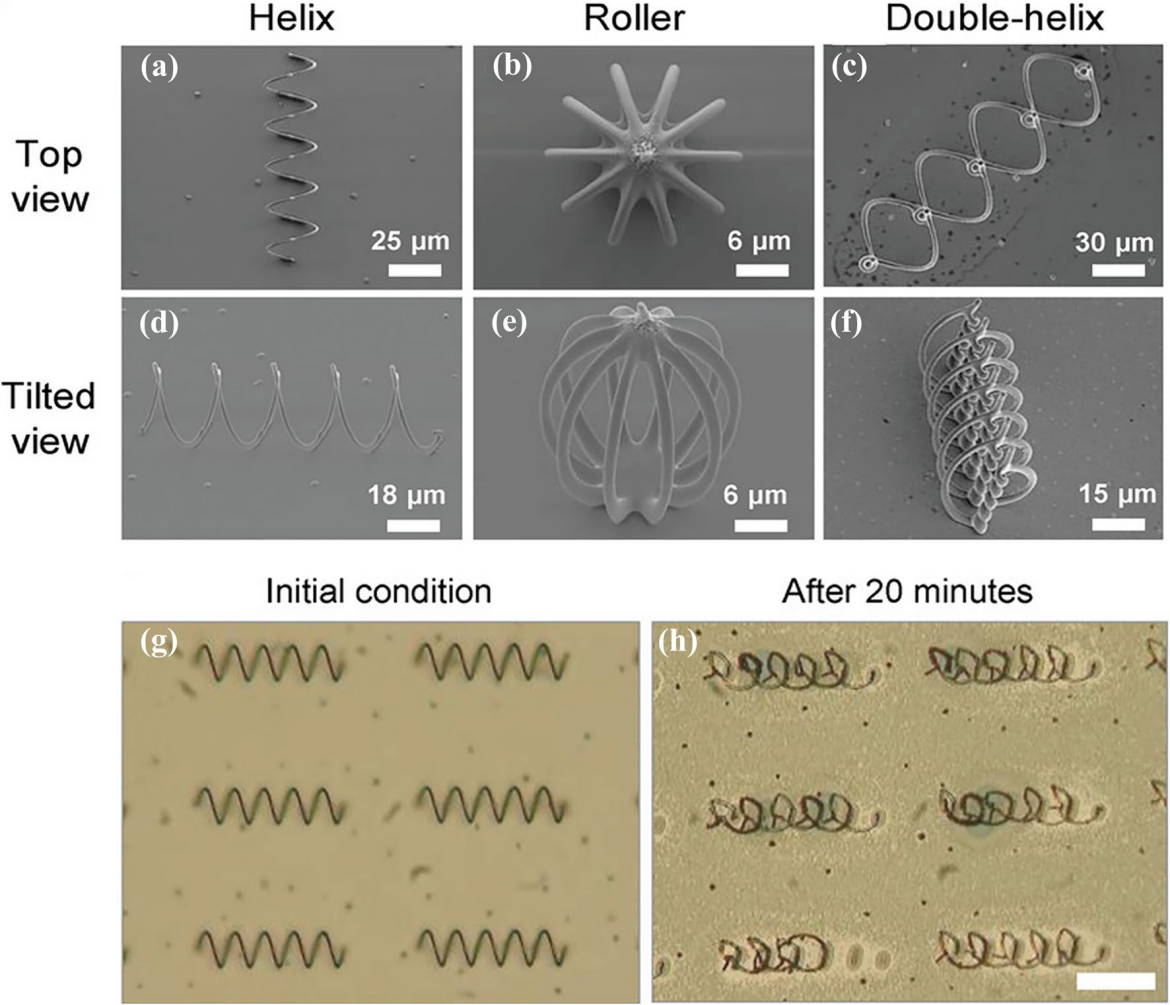
Scanning electron micrographs of 3D template-assisted electrodeposited iron-based (**a**) and (**d**) micro-helices, (**b**) and (**e**) micro-roller and (**c**) and **(f**) double helices. In vitro degradation of iron-based microrobot in simulated gastric fluid (**g**) before and (**h**) after 20 min of incubation. Scale bar 50 µm. Reproduced with permission [141]. Copyright 2019, Wiley publication

Alcantara et al. fabricated an Fe-based helical and roller microrobot by 3D template-assisted electrodeposition (Fig. 9a–f) [141]. In vitro degradation test showed that the micro-helices were degraded after 20 min of immersion in simulated gastric acid (Fig. 9g–h). According to live-dead imaging, Fe micro-helices did not exert a toxic effect on HCT116 cells after 4 days of incubation. The velocities of the micro-helix and scaffold-like micro-roller were compared under magnetic actuation. The micro-helix showed a maximum velocity of 42 µm s^−1^ at 10 mT and 7 Hz, whereas the porous micro-sphere exhibited a maximum velocity of 25 µm s^−1^ at 10 mT and 5 Hz in 100 cSt silicone oil. The upstream motion of the fully metallic Fe micro-helix and its Ni-coated polymer counterpart was measured in a microfluidic channel in water under a rotating magnetic field (20 mT and 30 Hz). Although both microstructures could swim at a velocity of 32 µm s^−1^under an applied magnetic field (20 mT and 30 Hz), the polymer structure exhibited unstable behavior. Therefore, a fully magnetic microstructure might be able to navigate human-vasculature regions with high flow velocities because of their generation of a high level of magnetic torque.

## Concluding Remarks and Future Directions

In the last decade, numerous research projects have developed degradable metallic micromotors as proofs of concept. Mg, Zn, and Fe are useful for degradable micromotors/robots because they react with water or physiological solutions, leading to complete degradation. To date, most degradable metallic micromotors use chemical reactions with water for propulsion. The lifetime of degradable metals can be tuned by functionalization and alloying. These materials have inherently excellent biocompatibility, including environmentally compatible degradation products. Also, degradable metals have potential as nutritional supplements, and their daily intake by human is high.

Degradable micromotors have a limited lifetime and uncontrolled autonomous motion. For example, the rapid degradation of Mg in physiological solutions leads to a shorter lifetime and can result in Mg being unable to efficiently perform its intended role. Therefore, the design of degradable micromotors is key to their propulsion and performance. To prolong the lifetime of degradable micromotors, the size and shape of the opening at its end must be optimized. Micromotors are used as drug carriers and can exert a therapeutic effect by generating hydrogen. However, hydrogen has low water solubility and can cause embolism. Hence, micromotors should be designed in such a way that they can be degraded in a controlled manner without causing adverse reactions.

Ions released from micromotors increase the local ion concentration, possibly resulting in detrimental effects. For example, excessive release of Mg ions leads to hyper-magnesia with side effects of nausea, low blood pressure, and low heart rate. An optimized design, including favorable surface modifications, is required for real-life application of degradable micromotors.

Mg micromotors degrade more rapidly than Zn and Fe micromotors/robots. Consequently, an exclusive design approach is necessary for Mg micromotors to extend their effective lifespan. By contrast, Zn and Fe exhibit relatively slow degradation rates due to their higher standard electrode potential than Mg. Hence, it is anticipated that Zn and Fe might show enhanced efficiency in carrying out their respective tasks. Nonetheless, the lower daily intake and biocompatibility might affect their implementation. To date, few studies have reported the potential of Zn and Fe-based micromotor/robots for biomedical and environmental applications. Therefore, future research may focus on new design strategies to uncover the potential of these two biodegradable metals for biomedical and environmental applications.

Surface functionalization of degradable robots may allow them to perform as smart materials. Therefore, next-generation degradable micromotors should incorporate smart materials to enhance their intelligence and performance. Present-generation degradable robots are propelled in an uncontrolled manner by chemical reactions of water/acid. For guided drug delivery or environmental remediation, a magnetic material (Ni, SPIONs) can be integrated to enable magnetic guidance. Furthermore, the magnetically guided lifetime of a degradable micromotor can be extended by full encapsulation with a degradable polymer, preventing initial rapid degradation. Therefore, the development of next-generation degradable micromotors is anticipated to have a marked impact in various fields, such as targeted drug delivery, diagnostics, and environmental remediation.
